# Molecular analysis of *Spiophanes bombyx* complex (Annelida: Spionidae) with description of a new species

**DOI:** 10.1371/journal.pone.0234238

**Published:** 2020-07-01

**Authors:** Vasily I. Radashevsky, Victoria V. Pankova, Vasily V. Malyar, Tatyana V. Neretina, Jin-Woo Choi, Seungshic Yum, Céline Houbin

**Affiliations:** 1 National Scientific Center of Marine Biology, Far Eastern Branch of the Russian Academy of Sciences, Vladivostok, Russia; 2 White Sea Biological Station, Faculty of Biology, Lomonosov Moscow State University, Moscow, Russia; 3 Kharkevich Institute for Information Transmission Problems, Russian Academy of Sciences, Moscow, Russia; 4 Research Institute of Oceanography, Seoul National University, Seoul, Republic of Korea; 5 Ecological Risk Research Division, Korea Institute of Ocean Science & Technology, Geoje, Republic of Korea; 6 Station Biologique de Roscoff, CNRS-Sorbonne Université, Roscoff, France; Sao Paulo State University (UNESP/FCL/Assis), BRAZIL

## Abstract

*Spiophanes bombyx* (Claparède, 1870) from the Gulf of Naples, Tyrrhenian Sea, Italy, was the first described *Spiophanes* with fronto-lateral horns on the prostomium. It was also considered the only horned species occurring in European waters. Our sequence data of five gene fragments suggest the presence of two horned sibling *Spiophanes* species in northern Europe: *S*. cf. *bombyx* in the North and the Norwegian seas, and *S*. cf. *convexus* in Brittany, northern France, and Bay of Biscay, northern Spain. *Spiophanes* cf. *bombyx* worms are genetically close to a single examined specimen of *S*. *bombyx* from Venice Lagoon, Italy but their conspecificity should be verified by further study. Our sequence data show that horned *Spiophanes* from the North Pacific are genetically distant from horned European species, and that *S*. *uschakowi* Zachs, 1933, originally described from the Sea of Japan (East Sea) is a valid species. The data also suggest the presence of two horned sibling *Spiophanes* species in the North East Pacific: *S*. *hakaiensis* Radashevsky & Pankova, n. sp. distributed from Alaska south to about Point Conception, and *S*. *norrisi* Meißner & Blank, 2009, distributed from San Francisco Bay south to Baja California Sur, Mexico. *Spiophanes* from South America, morphologically similar to *S*. *norrisi*, are suggested to belong to a new species. Molecular data also suggest the presence of two sibling species among the worms from northern Europe identified by morphology as *S*. *kroyeri* Grube, 1860. Worms from the Barents Sea and northern part of the North Sea are tentatively referred to as *S*. cf. *kroyeri*; worms from the northern and central parts of the North Sea and from the Bay of Biscay, northern Spain, are tentatively referred to as *S*. cf. *cirrata* M. Sars in G.O. Sars, 1872. Sequence data also show that *S*. *duplex* from California is genetically different from morphologically similar worms from South America. The South American worms are referred to resurrected *S*. *soederstroemi* Hartman, 1953 which was originally described from off Rio Grande do Sul, Brazil, and then considered as a junior synonym of *S*. *duplex*. Analysis of divergence times of *Spiophanes* lineages suggested that the origin of the most recent common ancestor of horned *Spiophanes* with metameric nuchal organs was around 11.1 mya (95% HPD: 5.1–19.0 mya) and that the divergence of the North Atlantic and North Pacific lineages was around 7.9 mya (95% HPD: 4.1–13.3 mya). The North Atlantic lineage was estimated to have diverged 4.8 mya (95% HPD: 2.2–8.6 mya), resulting in the origin of *S*. cf. *bombyx* and *S*. cf. *convexus*. The North Pacific lineage was estimated to have diverged first by the isolation and speciation of *S*. *norrisi* 1.7 mya (95% HPD: 2.3–1.0 mya), and then by the isolation and speciation of *S*. *uschakowi* and *S*. *hakaiensis* n. sp. 1.3 mya (95% HPD: 2.0–0.7 mya). The estimates place the divergences soon after maximum glacial period in the North Pacific (2.4–3.0 mya).

## Introduction

For a long time, polychaetes were believed to have high morphological variability and ecological plasticity and many species were considered as widely distributed or cosmopolitan. New approaches and techniques show, however, that many of these “cosmopolitans” comprise groups of similar cryptic or sibling species with much more limited variability, plasticity and geographic distribution than was previously suggested (see Read’s comments on Polychaeta in Appeltans *et al*. [1]; [2–4]).

Wide or cosmopolitan distribution has often been reported for members of one of the largest polychaete families, Spionidae Grube, 1850. Examples included *Aonides oxycephala* (Sars, 1862), *Boccardia polybranchia* (Haswell, 1885), *Dipolydora armata* (Langerhans, 1880), *Dipolydora coeca* (Örsted, 1843), *Dipolydora flava* (Claparède, 1870), *Dipolydora socialis* (Schmarda, 1861), *Laonice cirrata* (M. Sars, 1851), *Paraprionospio pinnata* (Ehlers, 1901), *Polydora ciliata* (Johnston, 1838), *Prionospio cirrifera* Wirén, 1883, *Prionospio steenstrupi* Malmgren, 1867, *Pygospio elegans* Claparède, 1863, *Scolelepis squamata* (Müller, 1806), *Spio filicornis* (Müller, 1776), and *Spiophanes bombyx* (Claparède, 1870). Common to all these species is that they were originally briefly described in the nineteenth century or even earlier. For example, the original description of *S*. *squamata* contains only three sentences in Latin [5]. Those original descriptions usually provided not specific but general characters shared also by other species from remote locations. Misidentification of those worms from remote locations incorectly “extended” the distribution of the stem species and created so-called “cosmopolitans due to incorrect identification”. It seems to be a destiny of old polychaete species to become cosmopolitans. Since most of the old species were originally described from Europe, consequently most of the cosmopolitan records are also European. Recent taxonomic revisions of *Laonice* Malmgren, 1867, *Paraprionospio* Caullery, 1914 and *Spiophanes* Grube, 1860 by Sikorski [6], Yokoyama [7] and Meissner [8], respectively, clarified some cases but uncertainties still remain.

One of the old *Spiophanes* species, *S*. *bombyx* was originally described from the Gulf of Naples, Italy, by Claparède [9, 10] (as *Spio bombyx*). The main diagnostic feature of the species was a pair of long horns extending from the fronto-lateral parts of the prostomium ([9]: Fig 2; [10]: Fig 2). Since then, *Spiophanes* with similar horns were reported as *S*. *bombyx* from northern Europe and Iceland [11–34, 8, 35], Atlantic coast of North and Central America [36–46], Atlantic coast of South America [47–52], Pacific coast of North America [53–66], Pacific coast of South America [67, 64, 68–73], Hawaii [74, 75], Pacific coast of Asia [76–95], Indian Ocean [96, 97], Atlantic coast of Africa [98–103], Australia and New Zealand [104–107], and Antarctica [108, 109]. Recent studies, however, suggested that these reports may include a series of cryptic species. Morphological examination of worldwide material and a molecular analysis (partial sequences of the nuclear *18S* rDNA and mitochondrial *COI*) confirmed the distribution of *S*. *bombyx* in the North and Mediterranean Seas and suggested that this species should not be considered as cosmopolitan [110, 8, 111].

Zachs [112] described *Spiophanes* with long fronto-lateral horns on the prostomium from Peter the Great Bay, Sea of Japan (East Sea), Russia, as *S*. *uschakowi* Zachs, 1933. The original description of the species was very brief and the type material is in poor condition [111]. Annenkova [113] suggested *S*. *uschakowi* to be a synonym of *S*. *bombyx* but Uschakov [77, 78] consistently treated the two species as different. Later authors reported from the Sea of Japan (East Sea) and adjacent areas only *S*. *bombyx* without comment on the taxonomy of *S*. *uschakowi* [76, 114, 83, 87, 88, 115]. Meißner & Blank [111] examined some earlier reports of *S*. *bombyx* from the American and Asian Pacific. They were uncertain about worms from the Asian Pacific because of the poor material available but, despite this, assigned specimens from the American Pacific to a new species, *S*. *norrisi* Meißner & Blank, 2009.

During systematic surveys on spionid polychaetes worldwide, we collected *Spiophanes* from the Atlantic and the Pacific Oceans, and the Mediterranean Sea. The main purpose of the present study was to clarify the specific identity of the horned *Spiophanes* from the North Pacific. Herein, we describe and illustrate adult morphology of *S*. *uschakowi* from the type locality in Peter the Great Bay, Russia, and use molecular analysis to provide new insight on the phylogenetic relationships among *Spiophanes* species.

## Materials and methods

### Collections, material and morphological study

**Fig 1:**
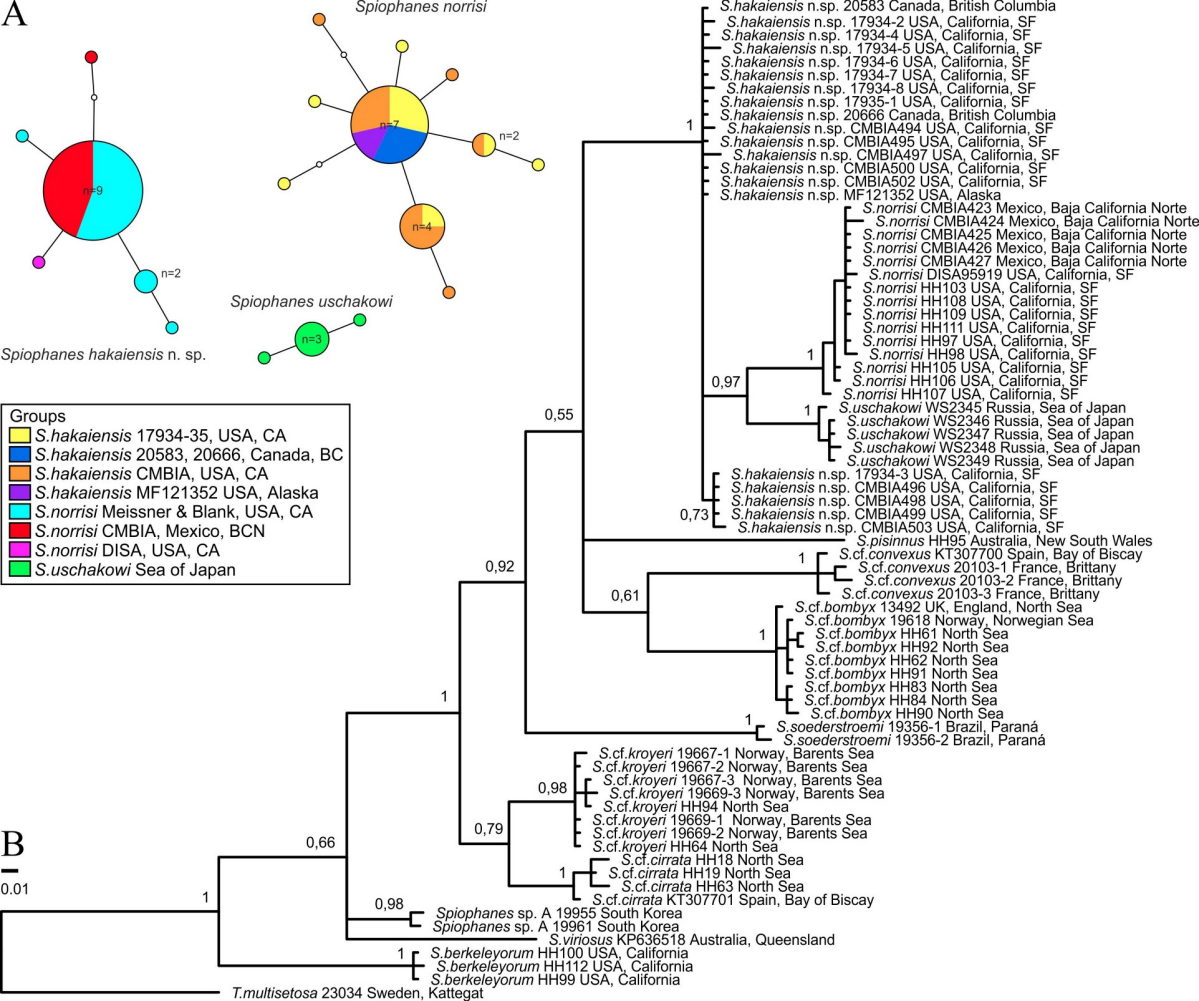
Phylogenetic results and haplotypes network. (A) Haplotype network of the *COI* (267 bp) sequences in *Spiophanes* spp. from North Pacific, calculated in TCS. Haplotypes are indicated by colored circles and their frequency is indicated by the size of the circles. Multiple colors indicate haplotypes shared by more than one sampling locality, with sections scaled by frequency. (B) Majority rule consensus tree of the Bayesian inference analysis-1 of *COI* (267 bp) sequences obtained in the present study and also taken from GenBank and BOLD. Posterior probabilities are shown on the branches. Species names are followed by the names of the collecting locations. Information about numbers with letters following specific names are given in Table 1. The numbers without letters following specific names are unique numbers from the VIR database linking the individuals on the tree with the sampling data in the S1 Table.

**Fig 2:**
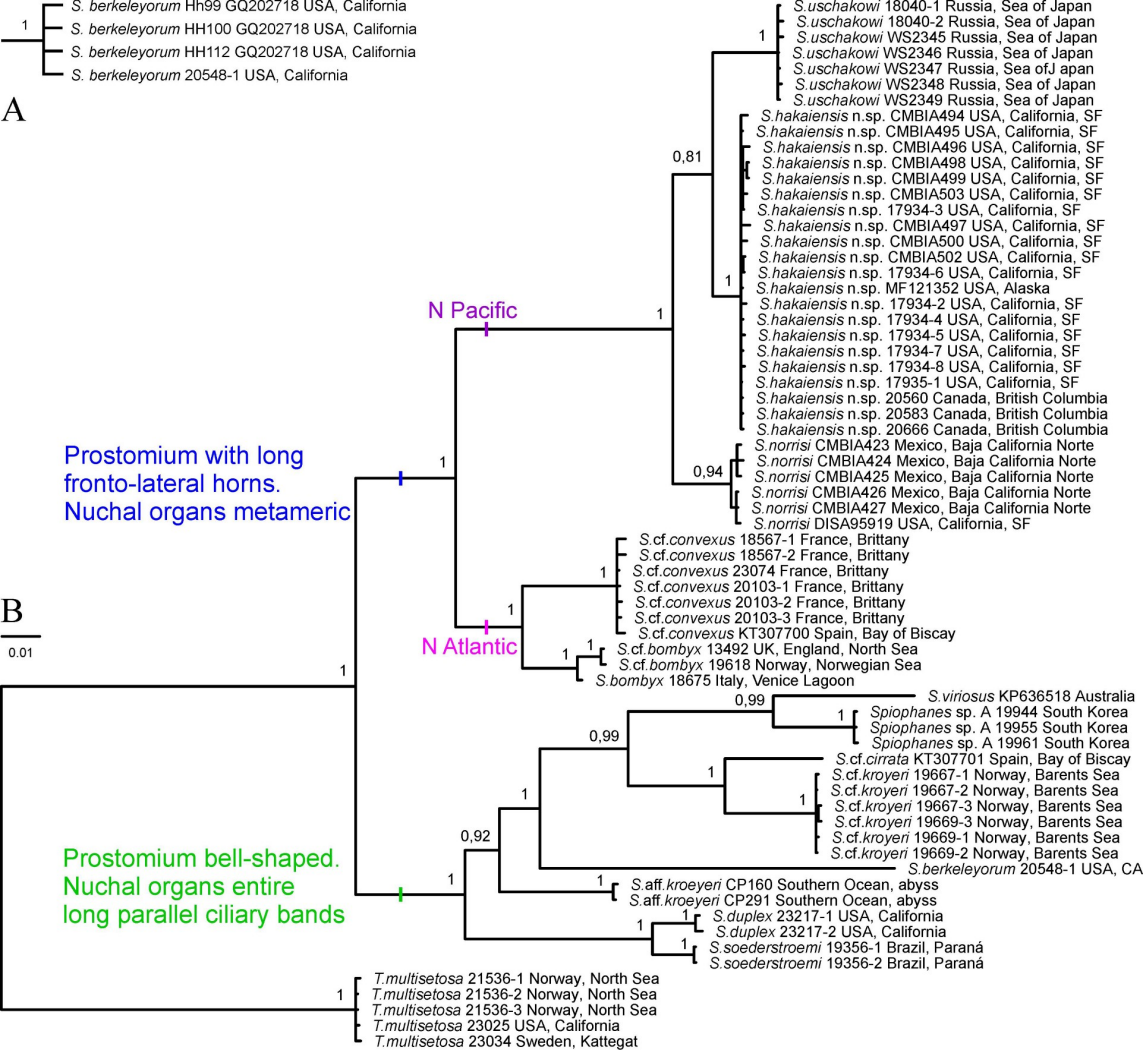
Phylogenetic results. (A) One branch from the Bayesian inference analysis of *18S* (406 bp) sequences obtained in the present study and provided by Meißner & Blank [111]. (B) Majority rule consensus tree of the Bayesian inference analysis of the combined *COI* (534 bp), *16S* (244 bp), *18S* (1656 bp), *28S* (287 bp), and *Histone 3* (297 bp) sequences (3018 bp in total) of *Spiophanes* spp. rooted with sequences of *Trochochaeta multisetosa*. Posterior probabilities are shown on the branches. Species names are followed by the names of the collecting locations. Information about numbers with letters following specific names are given in Table 1. The numbers without letters following specific names are unique numbers from the VIR database linking the individuals on the tree with the sampling data in the S1 Table.

**Fig 3:**
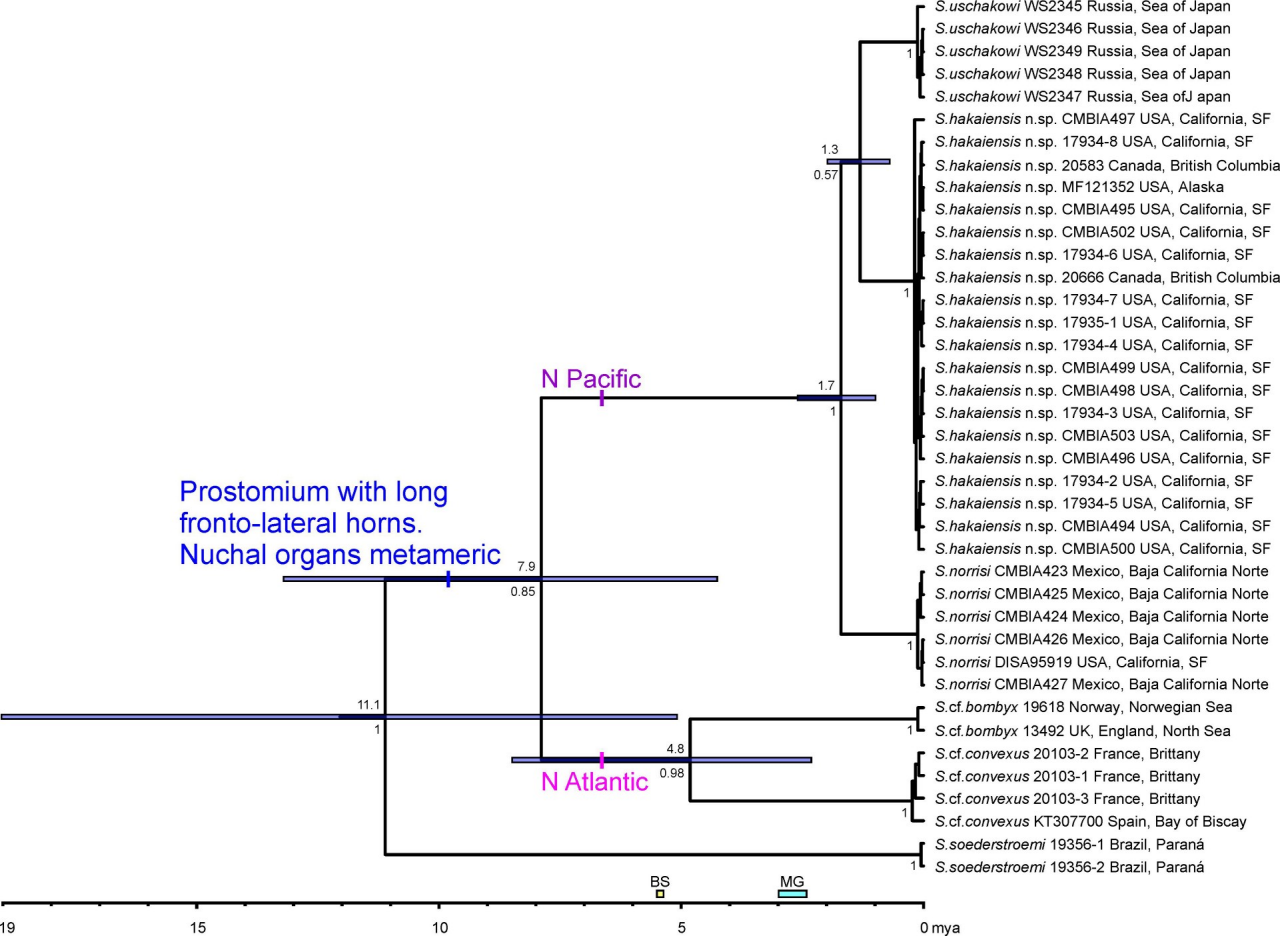
The maximum clade credibility chronogram of horned *Spiophanes* inferred by the BEAST analysis of *COI* sequences. Posterior probabilities are shown on the branches below the divergence time estimations. Bars at nodes indicate highest posterior density (95% HPD) intervals for the divergence times. Species names are followed by the names of the collecting locations. Information about numbers with letters following specific names are given in Table 1. The numbers without letters following specific names are unique numbers from the VIR database linking the individuals on the tree with the sampling data in the S1 Table. Abbreviations: BS–opening of the Bering Strait 5.4–5.5 mya; MG–maximum glacial period in North Pacific 2.4–3.0 mya.

Collections were made in shallow waters in the Sea of Japan (East Sea), Russia; around the Korean Peninsula; British Columbia, Canada; California, USA; Norwegian Sea, Norway; North Sea, Northumberland, UK; English Channel, Brittany, France; Venice Lagoon, Adriatic Sea, Italy; Chile, and Paranaguá Bay, Paraná, Brazil (Tables 1 and S1). Collected sediments were washed in the field on a 500 μm mesh sieve and *Spiophanes* worms retained in the residue were removed and examined alive under light microscopes in the laboratory. For molecular analysis, specimens were preserved in 95% ethanol. Voucher specimens were fixed in 10% formalin solution, rinsed in fresh water and then transferred to 70% ethanol. After examination, formalin preserved specimens were deposited in the polychaete collections of the Museum of the Institute of Marine Biology, National Scientific Center of Marine Biology (MIMB), Vladivostok, Russia; the White Sea Branch of the Zoological Museum of the Lomonosov Moscow State University, the White Sea Biological Station (WS), Poyakonda, Russia; Muséum National d’Histoire Naturelle (MNHNP), Paris, France; Senckenberg Museum (SMF), Frankfurt am Main, Germany; Natural History Museum of Los Angeles County (LACM-AHF), Los Angeles, CA, USA, and the United States National Museum of Natural History, Smithsonian Institution (USNM), Washington, D.C., USA. We additionally examined *Spiophanes* specimens deposited in the polychaete collections of the above mentioned museums and also in the Zoological Institute (ZISP), St Petersburg, Russia; Natural History Museum (NHMO), Oslo, Norway; Icelandic Institute of Natural History (IINH), Reykjavík, Iceland, and the California Academy of Sciences, Department of Invertebrate Zoology (CASIZ), San Francisco, CA, USA. Additional material of *Spiophanes* from the North Sea, Germany, was kindly provided by Dagmar Lackschewitz; from the Adriatic Sea, Croatia, by Barbara Mikac; and from Argentina by María Martha (Pitu) Mendez. Additional material of *S*. *norrisi* from California, USA, and *S*. *kroyeri* from Norway, originally preserved in ethanol, was kindly provided by Dorothy Norris and the University Museum of Bergen, Norway (ZMBN), respectively. Norwegian material was collected by the MAREANO Project. Material from British Columbia, Canada, was collected by the MarineGEO Hakai-Smithsonian BioBlitz-2017. Information about samples used for molecular analysis is given in Table 1. Complete available information about type specimens is given in the Results preceding the descriptions or comments on the species; information about all newly collected material and museum samples examined during this study is given in S1 Table. A complete list of the museums and other collections (and their acronyms) holding the examined or reported samples is given in S2 Table. S1 Table also includes material from the Norwegian, North, Mediterranean and Aegean seas and North Pacific reported by Meißner & Blank [111] and from the North Sea identified by Dieter Fiege, Markus Böggemann and Karin Meißner which is deposited in the SMF and other polychaete collections and was only partially re-examined by the first author (VIR). It also includes records of *S*. *bombyx* (= *S*. *uschakowi*) from Japan provided by Imajima [88, 116–118], for which no museum numbers were reported, records of *S*. *bombyx* (= *S*. cf. *norrisi*) from Chile, Argentina and the Falkland Islands provided by Carrasco [119] and Blake [48], and records of *S*. *bombyx* (here referred either to *S*. *bombyx* or *S*. cf. *convexus*) from Spain provided by Meißner [8]. To link some sequences used in the molecular analysis in the present study with the corresponding data, unique numbers from the first author’s database (VIR) are given to samples in the S1 Table (at the end of each record). These numbers without letters precede specific names on the phylogenetic trees (Figs 1–3).

**Table 1:** Sampling location data, museum registration numbers and GenBank accession numbers of sequences.

Species	Location	Coordinates	Date	Voucher^a^	GenBank/BOLD accession numbers^b^				
		Reference			*COI*	*16S*	*18S*	*28S*	*Histone 3*
1. ***Trochochaeta*** *multisetosa*	North Sea, Norway	58.24767°N, 6.53107°E	3 Feb 2016	ZMBN 126365		MN193552, 53		MN193539–41	MN193939–41
2. *T*. *multisetosa*	Askeröfjord, Sweden	58.1179°N, 11.828°E	11 May 2017	SIO BIC A6333	**MN313649**^c^		**MN296517**^c^		
3. *T*. *multisetosa*	Drake's Bay, California, USA	[120]	1 Apr 2003	VIR 23025			**DQ790097**	**DQ790070**	
4. ***Spiophanes*** *berkeleyorum*	offshore Long Beach, California, USA	33.66834°N, 118.29762°W	17 Jul 2017	MIMB 39031 (VIR 20548)			MN186816	MN193542	MN193942
5. *S*. *berkeleyorum*	offshore San Francisco, California, USA	[111]		HH99, 100, 112	**GQ202713, 14**		**GQ202718**		
6. *S*. *bombyx*	Adriatic Sea, Venice Lagoon, S. Andrea, Italy	45.4389°N, 12.39359°E	29 Jul 2014	MIMB 28156 (VIR 18675)		MG878899		MG878928	
7. *S*. cf. *bombyx*	North Sea (north, central and south-east parts)	[111]		HH61, 62, 83, 84, 90–92	**GQ202702–08**		**GQ202717**		
8. *S*. cf. *bombyx*	North Sea, Northumberland, England, UK	55.113°N, 1.413°W	19 Nov 2008	WS 2365 (VIR 13492)	KM998751	MG878898	KM998759	MG878927	MG874412
9. *S*. cf. *bombyx*	Norwegian Sea, Kvaløya Is., Norway	69.69104°N, 18.8737°E	11 Sep 2015	MIMB 28157 (VIR 19618)	MG874445	MG878900	MG913227	MG878929	MG874413
10. *S*. *duplex*	offshore Long Beach, California, USA	33.66834°N, 118.29762°W	17 Jul 2017	MIMB 39030 (VIR 23217)			MN186817	MN193543	MN193943, 44
11. *S*. cf. *convexus*	Bay of Biscay, Spain	[121]		VIR 23007	**KT307700**				
12. *S*. cf. *convexus*	Bay of Morlaix, Brittany, France	48.6502°N, 3.8651°W	27 May 2014	MIMB 28155 (VIR 18567)		MN193554		MN193544–46	MN193945
13. *S*. cf. *convexus*	Bay of Morlaix, Brittany, France	48.70572°N, 3.93395°W	6 Sep 2016	MIMB 36704 (VIR 20103)	MG874446−48	MG878901−03	MG913228−30	MG878930−32	MG874414−16
14. *S*. cf. *kroyeri*	Barents Sea, Norway	72.3075°N, 32.342667°E	4 Aug 2013	ZMBN 108407 (VIR 19667)	MG874451−53		MG913233−35	MG878935−37	MG874419−21
15. *S*. cf. *kroyeri*	Barents Sea, Norway	71.776833°N, 33.540333°E	7 Aug 2013	ZMBN 108408 (VIR 19669)	MG874454−56	MG878906, 07	MG913236−38	MG878938−40	MG874422−24
16. *S*. cf. *kroyeri*	North Sea	[111] as *S*. *kroyeri*		HH64, 94	**GQ202709, 10**		**GQ202719**		
17. *S*. cf. *cirrata*	North Sea	[111] as *S*. *kroyeri*		HH18, 19, 63	**GQ202696, GQ202711, 12**		**GQ202720**		
18. *S*. cf. *cirrata*	Bay of Biscay, Spain	[121] as *S*. *kroyeri*			**KT307701**				
19. *S*. aff. *kroyeri*	Crozet Islands, Southern Ocean, abyss	[122]		CP160, 291		**EU340080, 82**	**EU340094, 96**		
20. *S*. *norrisi*	offshore San Francisco, California, USA	[111]		HH97, 98, 103, 105–109, 111	**GQ202697–701**		**GQ202716**		
21. *S*. *norrisi*	offshore San Francisco, California, USA	37.8377°N, 122.62°W	11 Sep 2017	LACM:DISCO: 7567 (VIR 23045)	**DISA959–19**				
22. *S*. *norrisi*	Baja California Norte, Mexico	32.494°N, 117.163°W	26 Jan 2005	MBI-SCCWRP-556 (VIR 23044)	**CMBIA 423–427**				
23. *S*. *hakaiensis* n. sp.	Cook Inlet, Alaska, USA	59.468°N, 151.553°W	23 May 2011	11BIOAK-1443	**MF121352**				
24. *S*. *hakaiensis* n. sp.	British Columbia, Canada	51.6656°N, 128.0797°W	22 Jul 2017	LACM-AHF Poly (VIR 20560)		MN193555	MN186818	MN193547	MN193946
25. *S*. *hakaiensis* n. sp.	Calvert Is., British Columbia, Canada	51.644°N, 128.1195°W	24 Jul 2017	MIMB 36707 (VIR 20583)	MN520841	MN193556	MN186819	MN193548	MN193947
26. *S*. *hakaiensis* n. sp.	Calvert Is., British Columbia, Canada	51.6763°N, 128.1222°W	4 Aug 2017	Holotype CMNA 2019–0105 (VIR 20666)	MN414081	MN193557	MN186820	MN193549	MN193948
27. *S*. *hakaiensis* n. sp.	offshore San Francisco, California, USA	37.7125°N, 122.5648°W	13 Sep 2010	MIMB 39022 (VIR 17935)	MG874464	MG878914	MG913241	MG878948	MG874432
28. *S*. *hakaiensis* n. sp.	offshore San Francisco, California, USA	37.6588°N, 122.5615°W	15 Sep 2010	MIMB 39021 (VIR 17934)	MG874457−63	MG878908−13	MG913239, 40	MG878941−47	MG874425−31
29. *S*. *hakaiensis* n. sp.	offshore San Francisco, California, USA	37.6588°N, 122.5615°W	13 Sep 2010	MBI-SCCWRP-430 (VIR 22931)	**CMBIA 494–503**				
30. *S*. *pisinnus*	New South Wales, Australia	[111]		HH95	**GQ202715**		**GQ202721**		
31. *S*. *uschakowi*	Peter the Great Bay, Russia	42.89236°N, 132.73486°E	19 Oct 2011	MIMB 28164; WS 2345–2349 (VIR 17242)	KM998746−50	MG878915−19	KM998760−63	MG878949−53	MG874433−37
32. *S*. *uschakowi*	Peter the Great Bay, Russia	42.89236°N, 132.73486°E	15 Jul 2013	MIMB 28165 (VIR 18040)		MN193558, 59		MN193550, 51	MN193949, 50
33. *S*. *viriosus*	Queensland, Australia	[123]		AM W.44566	**KP636518**		**KP636519**		
34. *S*. *soederstroemi*	Paranaguá Bay, Paraná, Brazil	25.552°S, 48.415°W	9 Jul 2015	MIMB 28151 (VIR 19356)	MG874449, 50	MG878904, 05	MG913231, 32	MG878933, 34	MG874417, 18
35. *Spiophanes* sp. A	East China Sea, South Korea	33.5°N, 124.00°E	11 Nov 2015	MIMB 39027 (VIR 19944)		MG878920	MG913242	MG878954	MG874438
36. *Spiophanes* sp. A	East China Sea, South Korea	32.00°N, 125.00°E	2015	MIMB 39028 (VIR 19955)	MG874465		MG913243	MG878955	MG874439
37. *Spiophanes* sp. A	East China Sea, South Korea	33.00°N, 124.00°E	2015	MIMB 39029 (VIR 19961)	MG874466		MG913244	MG878956	MG874440

Sequences obtained in the present study are given in plain; sequences provided by other authors are given in **bold**.

^a^Vouchers by Meißner & Blank ([111]: Table 1) were cited as in their paper without explanation about HH abbreviation.

^b^Two last digits are shown for the second and other numbers in a successive series.

^c^Collected by Fredrick Pleijel, sequenced in Greg Rouse’ laboratory

When no coordinates were provided for sampling sites in old studies, they were collected from the Google Earth map according to the original descriptions of the locations. Sampling locations of *Spiophanes* spp. noted in S1 Table are plotted on maps using the QGIS 3.8.0 software and the geodata provided by the OpenStreetMap Project (https://osmdata.openstreetmap.de).

Formalin-fixed specimens of *S*. *uschakowi* were critical-point dried in carbon dioxide, coated with gold palladium, and viewed with a LEO 440 scanning electron microscope (SEM) equipped with a digital camera at the National Museum of Natural History, Smithsonian Institution, Washington, DC, USA.

Formalin-fixed specimens of *Spiophanes* spp. were stained with an alcohol solution of methyl green (MG), examined complete or dissected to observe internal structures, and photographed using microscopes equipped with digital cameras. Final plates were prepared using CorelDRAW®2017 software.

### DNA extraction, amplification and sequencing

We used the DNA Wizard Genomic DNA Purification Kit and the ReliaPrep gDNA Tissue Miniprep System (Promega Corporation, Madison, WI, USA) for DNA extraction and purification, with the standard protocol for animal tissue. Polymerase chain reaction (PCR) amplification of nuclear *18S* rDNA, D1 region of *28S* rDNA and *Histone 3*, and mitochondrial *16S* rDNA gene fragments was accomplished with the primers and conditions described by Radashevsky *et al*. [124, 125]. We used primers 5' GGTCAACAAATCATAAAGATATTGG 3' and 5' TAAACTTCAGGGTGACCAAAAAATCA 3' to amplify the mitochondrial gene fragment of c*ytochrome C oxidase subunit 1* (*COI*) [126]. Cycling parameters were as follows: initial denaturation at 94°C for 2 min, 35 cycles of 94°C for 30 s, 50°C for 40 s, 72°C for 60 s, with a final extension of 72°C for 5 min.

Purified PCR products were bidirectionally sequenced on an ABI Prism 3100 Genetic Analyzer (Applied Biosystems) using the BigDye Terminator v 3.1 Cycle Sequencing Kit (Applied Biosystems) and the same primers as for PCR. The consensus sequence of each gene region of each specimen was assembled from the two complementary sequences using SeqScape v 2.5 (Applied Biosystems). GenBank accession numbers of the obtained sequences are given in Table 1.

### Phylogenetic analysis

We aligned DNA sequences using the MAFFT v7.2 software with the default settings (automatically chosen algorithms) [127, 128]. Ambiguous positions and gaps were excluded from subsequent analysis using GBlocks [129]. The nucleotide datasets were combined using the supermatrix approach. The number of variable and parsimony informative sites in the datasets, the uncorrected values of sequence divergence (pairwise distances, *p*) both within and between groups were calculated in MEGA 5.1.

We used MrBayes 3.2.6 via the CIPRES web portal [130] for the Bayesian analysis of 10,000,000 generations, four parallel chains and sample frequencies set to 500, in two separate runs. Based on the convergence of likelihood scores, 25% sampled trees were discarded as burn-in. The analysis of the combined data set was partitioned and the models of substitution were determined using Akaike Information Criterion (AIC) in Modeltest 3.7 [131]: GTR+I+G for *16S*, *28S* and *Histone 3*, TVM+G for *18S*, and HKY+I+G for *COI*.

We also included in the analysis sequences of *Spiophanes* spp. obtained by Meißner & Blank [111], Mincks *et al*. [122], Meißner & Götting [123], Aylagas *et al*. [121], and some sequences from GenBank and BOLD (Table 1). We performed two analyses: the *COI* analysis to compare our data with those reported by previous authors, and a concatenated analysis using five genes to get better resolution of relationships among *Spiophanes* species. The five-genes analysis was based on sequence data of the mitochondrial *COI*, *16S* rDNA, nuclear *18S*, *28S* rDNA, and *Histone 3* most of which were obtained in the present study. In this analysis, we included *COI* sequences obtained by other authors if they comprised more than 500 bp, and *18S* sequences if they comprised more than 1600 bp. Of 46 individual concatenated sequences obtained in the present study, 30 sequences comprised fragments of four to five genes, and 39 sequences comprised fragments of three genes. The analyses were rooted using the sequences of *Trochochaeta multisetosa* (Örsted, 1843). In the Bayesian analysis of *18S* sequences of various spionids by Mincks *et al*. ([122]: Fig 8), *Trochochaeta* Levinsen, 1883 appeared sister to *Spiophanes*.

The haplotype network was constructed based on a statistical parsimony approach using TCS [132].

### Divergence time estimation

Divergence time estimation of the horned *Spiophanes* lineages was performed with BEAST 1.8.0 [133] running in the CIPRES Science Gateway [130]. For the analysis, we used *COI* sequences comprising more than 500 bp. The in-group included horned *Spiophanes* with metameric nuchal organs, all members of a monophyletic group according to the five-genes analysis. The analysis was rooted using the sequences of *S*. *soederstroemi* that in five-genes analysis appeared most closely related to the clade of the horned *Spiophanes*.

The Tamura-Nei model (TrN) of nucleotide substitution with gamma-distributed rate variation (+G) was applied to *COI* data set with base frequencies estimated during the analysis [134]. We used the path sampling maximum likelihood estimator implemented in BEAST 1.8.0 [135, 136] to determine the appropriate molecular clock models for data set (uncorrelated lognormal relaxed clock, random local clock or strict clock). After model selection, a relaxed molecular clock using the uncorrelated log-normal model [137] was applied with a Birth-Death process speciation prior for branching rates [138]. We applied substitution rate of 4.1%/MY with a SD of 3.5–4.7%/MY, suggested for northern polychaetes by Loeza-Quintana *et al*. [139], to convert genetic branch lengths to absolute times. The final analysis consisted of two independent MCMC analyses; each chain was run for 40,000,000 generations with parameters sampled every 4000 steps to ensure effective sample size (ESS) values above 200 for all parameters. The first 10% of trees in each independent run were discarded as burn‐in and the remaining trees were combined to produce an ultra‐metric consensus tree using LogCombiner and TreeAnnotator 1.8.0 (BEAST package).

### Nomenclatural acts

The electronic edition of this article conforms to the requirements of the amended International Code of Zoological Nomenclature, and hence the new names contained herein are available under that Code from the electronic edition of this article. This published work and the nomenclatural acts it contains have been registered in ZooBank, the online registration system for the ICZN. The ZooBank LSIDs (Life Science Identifiers) can be resolved and the associated information viewed through any standard web browser by appending the LSID to the prefix “http://zoobank.org/”. The LSID for this publication is: urn:lsid:zoobank.org:pub:B69BEC0A-2B46-4F46-99E4-73FD7D502A38. The electronic edition of this work was published in a journal with an ISSN, and has been archived and is available from the following digital repositories: PubMed Central, LOCKSS, ResearchGate.

## Results

### Molecular analysis

#### *COI* analysis and haplotype network

The alignment of *COI* sequences obtained in the present study and those from GenBank and BOLD comprised 267 bp with 106 (39.7%) variable sites, of which 93 (34.8%) sites were parsimony informative. The Bayesian analysis resulted in a partially resolved consensus tree (Fig 1B).

The analysis revealed two sister groups among specimens from northern Europe identified by morphology as *S*. *bombyx*: one from the North and the Norwegian seas (referred to as *S*. cf. *bombyx*), and another from Brittany, northern France, and the Bay of Biscay, northern Spain (referred to as *S*. cf. *convexus*). The *p-*distances among individuals of these groups ranged from 12.73% to 14.98%. The ingroup *p-*distance values ranged from 0.00% to 1.5% and from 0.75% to 1.87%, respectively.

Horned *Spiophanes* from the North Pacific formed a well supported clade (PP = 1) closely related to the clade of horned *Spiophanes* (*S*. cf. *bombyx*—*S*. cf. *convexus*) from northern Europe. The relationships within the North Pacific clade were partially resolved, with one clade well supported (PP = 0.97), comprising one group of specimens from California, USA, and Baja California Norte, Mexico (referred to as *S*. *norrisi* according to the type locality of the species in Baja California Sur, Mexico), and another group *S*. *uschakowi* from the Sea of Japan (East Sea). The *p-*distances between individuals of these groups ranged from 6.74% to 8.99%. The rest of the specimens within the North Pacific clade did not form a monophyletic group, but, according to the results of the five-genes analysis (see below), they are referred to *S*. *hakaiensis* n. sp. The three groups of North Pacific *Spiophanes* did not share any haplotype (Fig 1A).

Two sister groups were revealed among specimens from northern Europe identified by morphology as *S*. *kroyeri*: one from the Barents Sea and northern part of the North Sea (tentatively referred to as *S*. cf. *kroyeri*), and another from the northern and central parts of the North Sea and from Bay of Biscay, northern Spain (tentatively referred to as *S*. cf. *cirrata*). The *p*-distances between individuals of these groups ranged from 6.37% to 8.61%. The ingroup *p*-distance values ranged from 0.00% to 1.12% and from 0.75% to 1.5%, respectively.

#### Five-genes analysis (*COI*, 16S, 18S, 28S, and Histone 3)

The five-genes analysis did not include sequences provided by Meißner & Blank [111] because they were too short and did not fit the accepted criteria (*COI* > 500 bp, *18S* > 1600 bp). The combined alignment, with gaps excluded, comprised in total 3018 bp, including 244 bp (93.5%) for *16S* rDNA, 1656 bp (99%) for *18S* rDNA, and 287 bp (98.6%) for *28S* rDNA, 297 bp (100%) for *Histone 3* and 534 bp (100%) for *COI*. It contained 677 (22.4%) variable sites, of which 615 (20.4%) sites were informative. The average *p-*distances for the individual gene fragments between taxa are given in S3–S7 Tables. The Bayesian analysis resulted in a fully resolved tree with high support of all branches (Fig 2B).

The analysis revealed two sister groups among specimens from northern Europe identified by morphology as *S*. *bombyx*. Specimens from the North and the Norwegian seas (referred to as *S*. cf. *bombyx*) were genetically close to single examined specimen of *S*. *bombyx* from Venice Lagoon, Adriatic Sea, Italy (*p*-distances ranging from 00% for *28S* to 2.46% for *16S*). Specimens from Brittany, northern France, and the Bay of Biscay, northern Spain, are referred to as *S*. cf. *convexus*. The *p-*distances between individuals of *S*. cf. *bombyx* and *S*. cf. *convexus* ranged from minimal 0.3% for *18S* rDNA to maximal 14.07% for *COI*. The maximum ingroup *p-*distance values were for *COI* sequences: 0.94% and 1.69%, respectively.

Horned *Spiophanes* from the North Pacific formed three distinct groups within a clade sister to the European horned *Spiophanes*. The groups comprised *S*. *uschakowi* from the Sea of Japan (East Sea), worms from Alaska to San Francisco Bay, California, described below as *S*. *hakaiensis* n. sp., and worms from San Francisco Bay, California, and Baja California Norte, Mexico, referred to *S*. *norrisi*. *Spiophanes uschakowi* and *S*. *hakaiensis* n. sp. formed a clade sister to *S*. *norrisi*. The *p-*distances between individuals of *S*. *uschakowi* and *S*. *hakaiensis* n. sp. ranged from minimal 0.91% for *18S* rDNA to maximal 7.68% for *COI*. The maximum ingroup *p-*distance values were for *COI* sequences: 0.94% and 1.12%, respectively.

Three groups were distinguished among specimens identified by morphology as *S*. *kroyeri*: one from the Barents Sea and northern part of the North Sea (referred to as *S*. cf. *kroyeri*), second from the northern and central parts of the North Sea and from the Bay of Biscay, northern Spain (referred to as *S*. cf. *cirrata*), and a third from the Southern Ocean (referred to as *S*. aff. *kroyeri*). The *S*. cf. *kroyeri* group appeared sister to *S*. cf. *cirrata*. The average *p-*distances for the individual gene fragments between these groups are given in S3–S7 Tables. *Spiophanes* aff. *kroyeri* appeared distant from the clade *S*. *kroyeri*—*S*. cf. *cirrata*.

Two sister groups were revealed among specimens identified by morphology as *S*. *duplex*: one from California, USA (referred to as *S*. *duplex*), and another from Paraná, Brazil (referred to as *S*. *soederstroemi*). The *p*-distances between individuals of these groups ranged from 0.18% for *18S* to 5.05% for *Histone 3*.

Horned *Spiophanes* with metameric nuchal organs formed a clade sister to the clade of *Spiophanes* with bell-shaped prostomium and nuchal organs as a pair of long parallel ciliary bands extending over about 15 anterior chaetigers (Fig 2B).

The analysis showed identity (*p-*distance = 0.00%) of *18S* and *28S* rDNA sequences of *Trochochaeta multisetosa* from Norway, Sweden and California, USA.

An additional analysis showed identity (*p-*distance = 0.00%) of our sequence of *S*. *berkeleyorum* from Southern California and short fragments of *18S* rDNA (490 bp) of *S*. *berkeleyorum* from Northern California obtained by Meissner & Blank [111] (Fig 2A).

#### Divergence time estimation

The alignment of *COI* sequences obtained in the present study and those from GenBank and BOLD comprised 534 bp with 170 (31.8%) variable sites, of which 160 (94.1%) sites were parsimony informative. The BEAST analysis resulted in a fully resolved consensus tree (Fig 3) with the same topology as the tree from the five-genes Bayesian analysis (Fig 2B). The analysis suggested that the origin of the most recent common ancestor of horned *Spiophanes* with metameric nuchal organs was around 11.1 mya (95% HPD: 5.1–19.0 mya) and that the divergence of the North Atlantic and North Pacific lineages was around 7.9 mya (95% HPD: 4.1–13.3 mya). The North Atlantic lineage was estimated to have diverged 4.8 mya (95% HPD: 2.2–8.6 mya), resulting in the origin of *S*. cf. *bombyx* and *S*. cf. *convexus*. The North Pacific lineage was estimated to have diverged first by the isolation and speciation of *S*. *norrisi* 1.7 mya (95% HPD: 2.3–1.0 mya), then by the isolation and speciation of *S*. *uschakowi* and *S*. *hakaiensis* n. sp. 1.3 mya (95% HPD: 2.0–0.7 mya).

### Morphology and biology

#### *Spiophanes bombyx* (Claparède, 1870)

*Spio bombyx* Claparède, 1870a [9]: 485–487, pl XII, Figs 2, 2A–2H; [10]: 121–123, pl XII, Figs 2, 2A–2H. *Fide* Mesnil [11].

*Spiophanes bombyx*: Fauvel [140] (*Part*.): 41. Rullier [141]: 209. Bhaud [142]: 235; [143]: 290–291, 306 (larval phenology). Katzmann [144]: 133. Cognetti-Varriale & Zunarelli-Vandini [145]: 42. Gambi & Giangrande [146]: 851. Sardá [147]: 346. Somaschini [148]: 223. Martin *et al*. [149]: 5. Simboura & Nicolaidou [150]: 87. Aleffi *et al*. [151]: 221. Ayari & Afli [152]: 89; [153]: 73. Dagli *et al*. [154]: 155. Castelli *et al*. 20[155]08: 357. Meißner [8] (*Part*.): 54–58. Meißner & Blank [111] (*Part*.): 7–11, Figs 3 and 4C. Mutlu *et al*. [156]: 32. Lorenti *et al*. [157]: 331. Serrano *et al*. [158]: 339. Çinar *et al*. [159]: 2114; [160]: 1462. Mikac [161]: 123. Delgado-Blas *et al*. [162] (*Part*.): 340.

*Type material*. ITALY, Tyrrhenian Sea, Gulf of Naples, presumably 40.8287°N, 14.2474°E, types lost.

*Synopsis*. Up to 38 mm long, 1 mm wide for 120 chaetigers. Prostomium with long fronto-lateral horns, posteriorly pressed into chaetiger 1 but not extending over it as caruncle. Occipital antenna absent. Nuchal organs metameric, on a series of anterior chaetigers; first pair of metamers as oblique ciliary bands from prostomium to end of chaetiger 2; succeeding metamers shorter. Sabre chaetae in neuropodia from chaetiger 15. Hooks in neuropodia from chaetiger 15, usually quadridentate, with small subterminal hood. Pygidium with small ventral fleshy pad and one pair of lateral cirri. Glandular organs in chaetigers 5–14, largest in chaetigers 7 and 8, smallest in chaetiger 9, gradually increasing in size from chaetiger 10 to chaetigers 12–13, slightly smaller again in chaetiger 14. Internal fibres long and coiled in glandular organs in chaetigers 5–8, straight, shorter and thinner in chaetigers 9–14. Organs on chaetigers 5, 7 and 8 each opening to exterior via semicircular to suboval slit around large fiber spreader, on chaetiger 6 via small round hole, on chaetigers 9–14 via large vertical slit. Frontal edge of each fiber spreader on chaetigers 5, 7 and 8 entire, rounded to blunt, or with variously developed middle depression vaguely separating two rounded lobes. Digestive tract with gizzard-like structure. Main dorsal blood vessel with heart body. Nephridia from chaetiger 15 onwards. Gonochoristic. Oocytes lentiform, each with honeycombed envelope and 18–20 cortical alveoli regularly arranged in a peripheral circle. Spermatids interconnected in tetrads. Spermatozoa short-headed aqua-sperm with plate-like acrosomes. Fertilization in sea water. Larval development holopelagic, planktotrophic. Larvae with two pairs of red eyes on prostomium and a midventral ciliated pit on chaetiger 2.

*Remarks*. In the original description of *S*. *bombyx*, Claparède ([9]: 485, as *Spio bombyx*) noted that “It does not seem rare in Naples. At least, it lives in numerous societies. I received hundreds of them one day, lodged side by side in tubes of black mud.” He reported worms up to 38 mm long, 1 mm wide. The type material of *S*. *bombyx* does not exist any longer, probably because Claparède did not deposit the examined specimens at any museum. After Claparède, the species has rarely been reported from Italy and seems not to be very common in the Gulf of Naples at present. We were unable to find a sequence of *S*. *bombyx* from the Mediterranean in the literature. We collected one individual of *S*. *bombyx* from Venice Lagoon, Italy, and sequenced it for *16S* and *28S* gene fragments (see Table 1).

Morphological examination and molecular analysis of horned *Spiophanes* performed in the present study discovered two sibling species in northern European waters which we refer to as *S*. cf. *bombyx* and *S*. cf. *convexus*. Their morphology, taxonomy and the distribution are discussed below. It seems plausible that *S*. *bombyx* does not occur in northern European waters and that its distribution is limited to the Mediterranean only. To avoid confusion in identification of specimens based on morphological characters only, especially in areas of possible overlap of the species, and until more molecular data has been obtained from specimens from the Gulf of Naples, we suggest that northern European horned *Spiophanes* are referred to as *S*. *bombyx* aggregate (*S*. *bombyx* agg.), rather than to a particular species.

**Fig 4:**
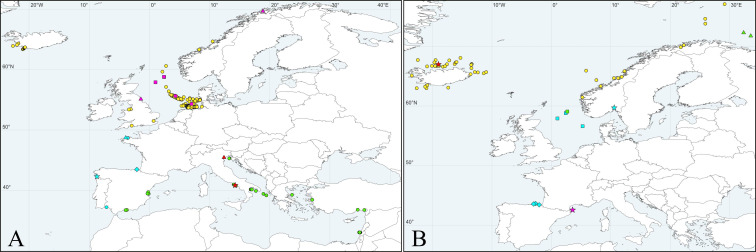
Maps showing records of *Spiophanes* spp. in North Atlantic Ocean and Mediterranean Sea. (A) ***Spiophanes bombyx*** (Claparède, 1870) (red and green symbols): red star–type locality: Gulf of Naples, Tyrrhenian Sea, Italy; red triangle–single specimen sequenced in the present study; green circles–adults identified based on the morphology only. ***Spiophanes* cf. *bombyx*** (yellow and pink symbols): pink triangles–specimens sequenced in the present study; pink squares–specimens sequenced by Meißner & Blank [111]; yellow circles–adults identified based on the morphology only. ***Spiophanes convexus*** (turquoise symbols): turquoise star–type locality of *S*. *convexus*: Meira, Pontevedra, Galicia, Spain; turquoise rhomb–*S*. cf. *convexus* sequenced by Aylagas *et al*. ([121], as *S*. *bombyx*); turquoise triangles–***S*. cf. *convexus*** sequenced in the present study; turquoise circles–adults identified based on the morphology only. (B) ***Spiophanes kroyeri*** Grube, 1860 (red, green and yellow symbols): red star–type locality: Greenland Sea, off Iceland; yellow circles–adults identified based on the morphology only; green triangles–***S*. cf. *kroyeri*** sequenced in the present study; green square–***S*. cf. *kroyeri*** sequenced by Meißner & Blank ([111], as *S*. *kroyeri*). ***Spiophanes cirrata*** M. Sars in G.O. Sars, 1872 (turquoise symbols): turquoise star–type locality: Drøbak, Oslofjord, Norway; turquoise squares–***S*. cf. *cirrata*** sequenced by Meißner & Blank ([111], as *S*. *kroyeri*); turquoise rhombs–***S*. cf. *cirrata*** sequenced by Aylagas *et al*. ([121], as *S*. *kroyeri*). ***Spiophanes reyssi*** Laubier, 1964: pink star–type locality: NE Lacaze-Duthiers, Gulf of Lion, Mediterranean France.

*Distribution*. Mediterranean Sea (Fig 4A).

**Fig 5:**
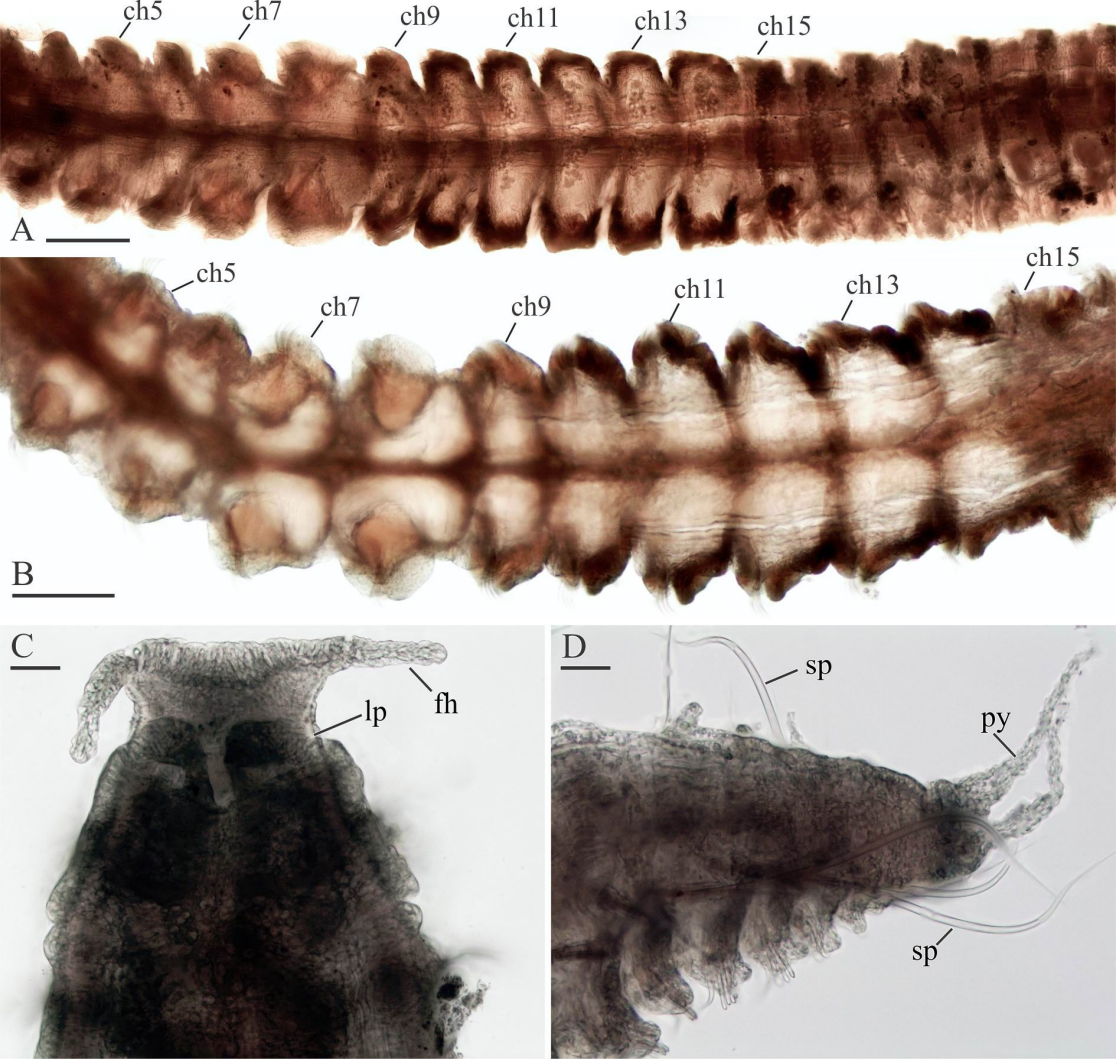
Adult morphology of *Spiophanes* cf. *bombyx* (Norway, MIMB 36701). (A), Chaetigers 4–20, dorsal view, showing light brownish lateral sides of chaetigers 9–14. (B) Chaetigers 5–16, ventral view, showing arrangement of glandular organs in chaetigers 5–14 and light brownish lateral sides of chaetigers 10–14. (C) Anterior end, ventral view. (D) Posterior end, left lateral view. Abbreviations: *ch5*–*ch15* –chaetigers 5–15; *fh*–fronto-lateral horn; *lp*–lateral peristomial lip; *py*–pygidial cirrus; *sp*–notopodial spine with recurved distal end. Scale bars: A– 300 μm; B– 200 μm; C, D– 50 μm.

**Fig 6:**
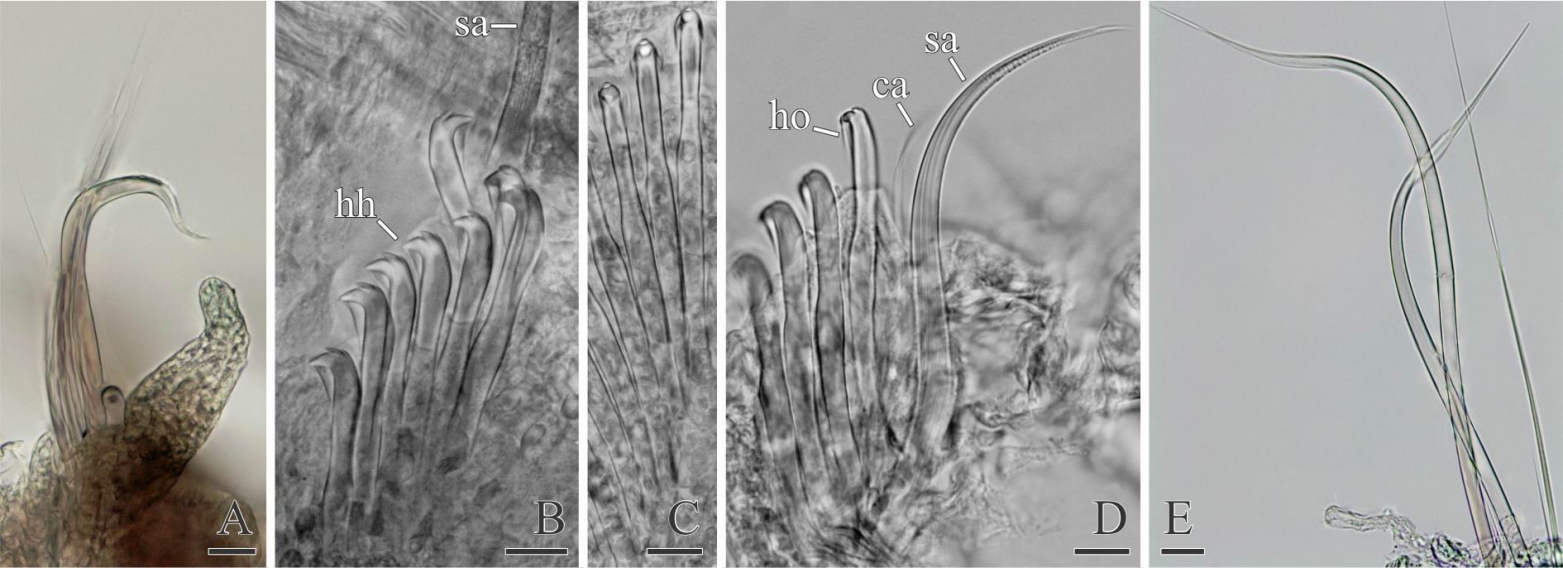
Chaetal morphology of *Spiophanes* cf. *bombyx* (Norway, MIMB 36701). (A) Crook-like spine in neuropodium of chaetiger 1. (B) Hooded hooks and a fragment of sabre chaeta from neuropodium of a middle chaetiger, lateral view. (C) Hooded hooks from neuropodium of a middle chaetiger, frontal view. (D) Hooded hooks, hair-like alimbate capillary chaeta, and a sabre chaeta from neuropodium of a posterior chaetiger, lateral view. (E) Simple capillary chaeta and two spines with recurved distal ends from posterior notopodia. Abbreviations: *ca*–capillary chaeta; *hh*–hooded hook; *ho*–subdistal hood below main fang of hook; *sa*–ventral inferior sabre chaeta. Scale bars: A, E– 20 μm; B–D– 10 μm.

**Fig 7:**
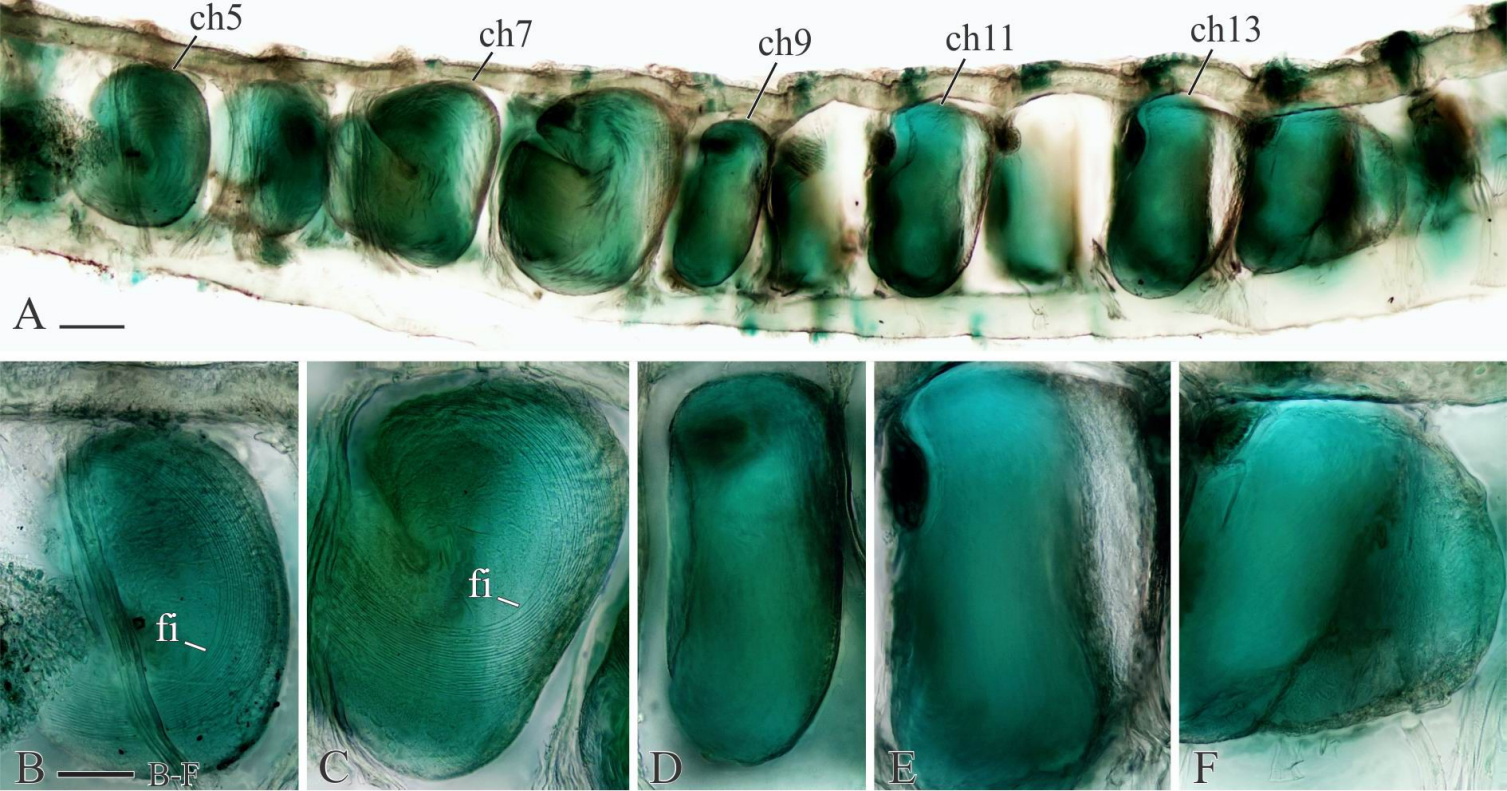
Glandular organs of *Spiophanes* cf. *bombyx* (Norway, MIMB 36701). Right-side organs in left lateral view; formalin-fixed specimen stained with an alcohol solution of methyl green. (A) arrangement of glandular organs in chaetigers 5–14; organs withdrawn from chaetigers 10 and 12. (B) Chaetiger 5. (C) Chaetiger 7. (D) Chaetiger 9. (E) Chaetiger 13. (F) Chaetiger 14. Abbreviations: *ch5*–*ch13* –chaetigers 5–13; *fi*–fibers produced by glandular organs. Scale bars: A– 100 μm; B–F– 50 μm.

**Fig 8:**
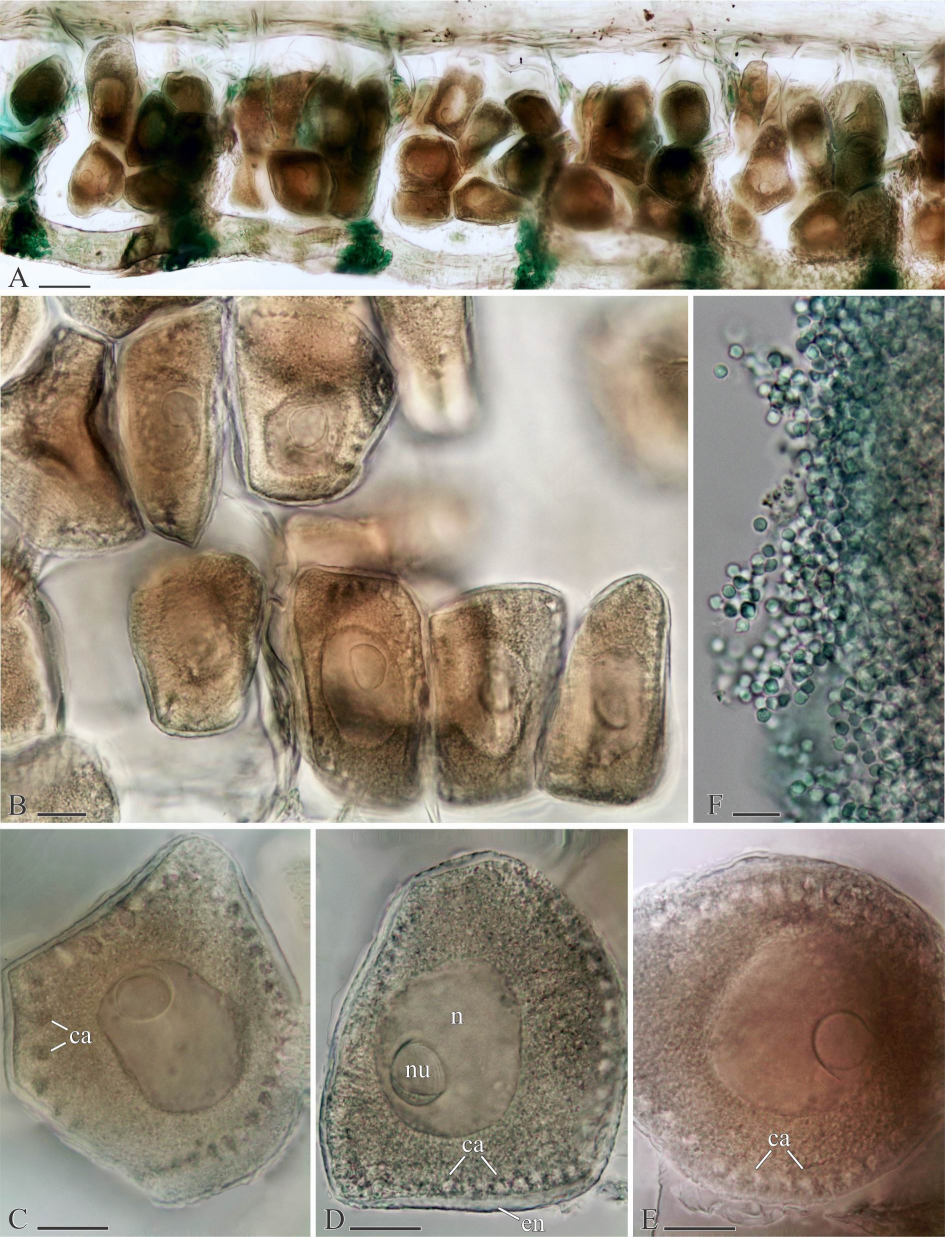
Gametes of *Spiophanes* cf. *bombyx* (Norway, MIMB 36701). Formalin-fixed specimens stained with an alcohol solution of methyl green. (A) Oocytes in chaetigers 21–25; right-side coeloms in left lateral view. (B) Coelomic oocytes in chaetigers 20 and 21. (C–E) Coelomic oocytes of different shape. (F) Coelomic spermatozoa. Abbreviations: *ca*, cortical alveoli; *en*, oocyte envelope; *n*, nucleus; *nu*, nucleolus. Scale bars: A– 300 μm; B–E– 20 μm; F– 10 μm.

***Spiophanes* cf. *bombyx* (Claparède, 1870).** Figs 5–8.

**Fig 9:**
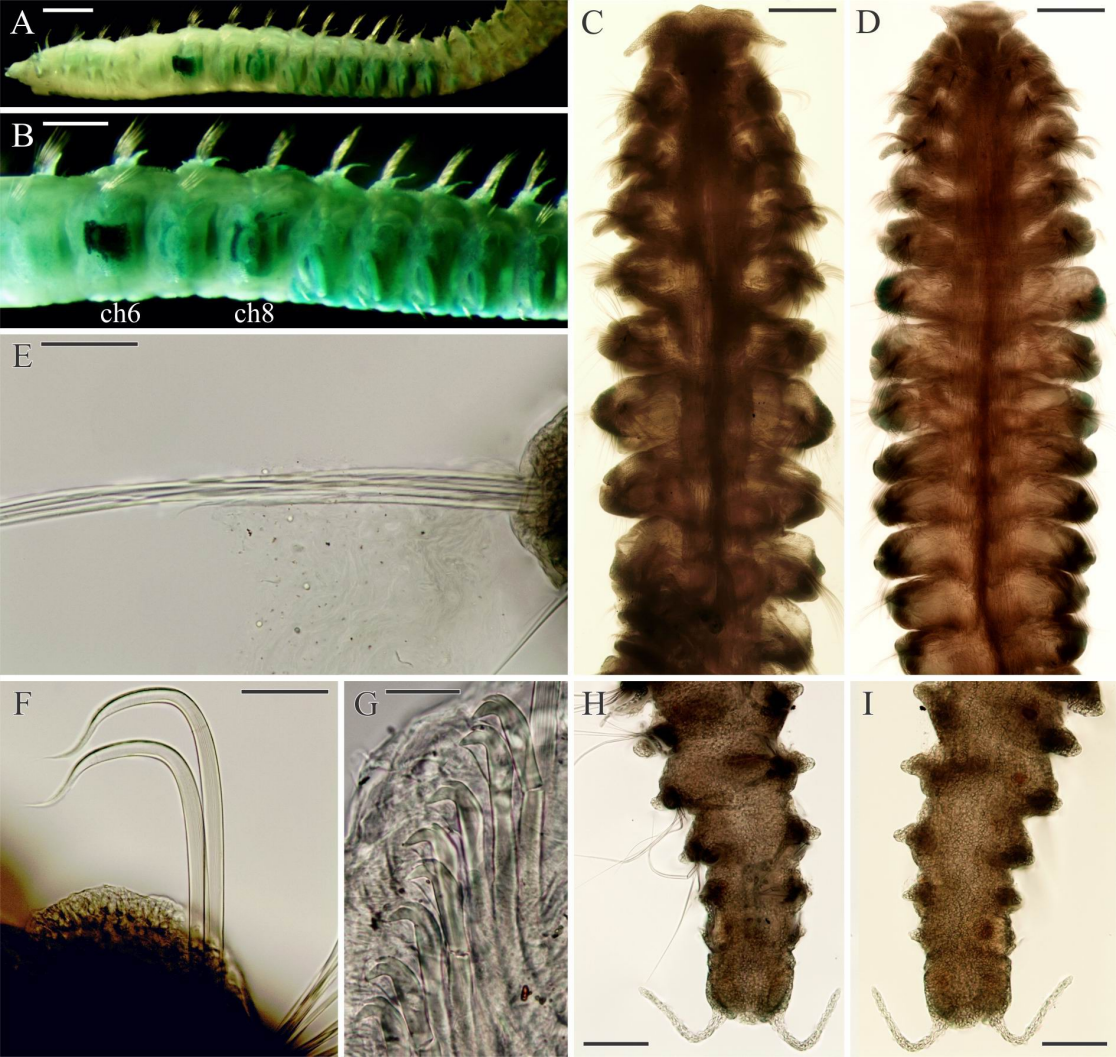
Adult morphology of *Spiophanes* cf. *convexus* (Brittany, France, MIMB 28155). (A, B) Formalin-fixed specimens stained with an alcohol solution of methyl green; palps missing. (A) Anterior end, left lateral view. (B) Chaetigers 5–13, left lateral view, showing specific pattern of methyl green staining. (C, D) Anterior ends, dorsal view, showing various shape of prostomium; palps missing. (E) Long thick fibers produced by glandular organ of chaetiger 6, protruding from hole-like opening of the organ. (F) Crook-like spines in neuropodium of chaetiger 1. (G) Hooded hooks from neuropodium of a middle chaetiger. (H) Posterior end, dorsal view. I, same, ventral view. Abbreviations: *ch6*, *ch8* –chaetigers 6 and 8; *fh*–fronto-lateral horn; *hh*–hooded hook; *lp*–lateral peristomial lip; *sa*–ventral inferior sabre chaeta. Scale bars: A– 500 μm; B–D– 300 μm; E, F– 50 μm; G– 20 μm; H, I– 100 μm.

**Fig 10:**
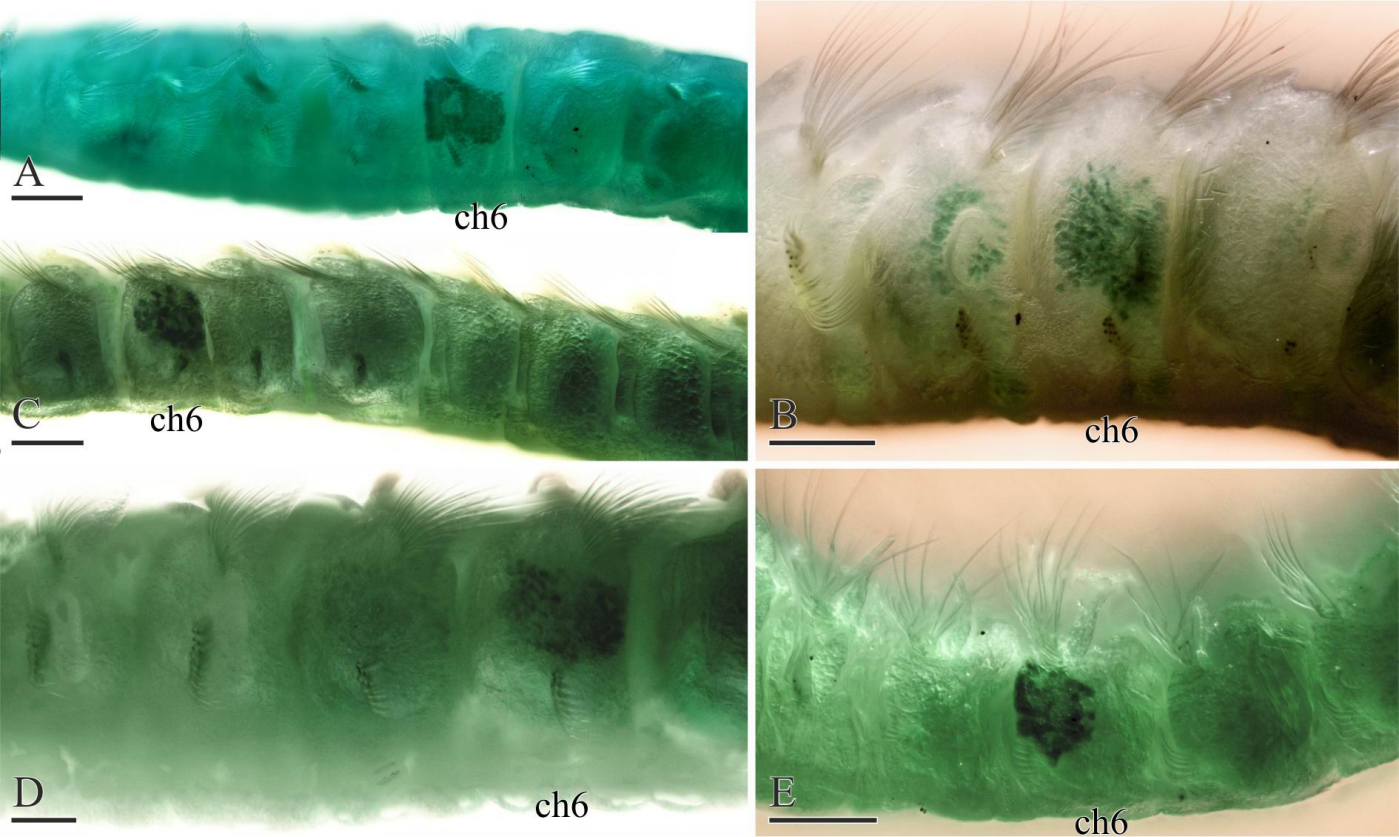
Methyl green staining patterns in formalin-fixed specimens of *Spiophanes* spp. (A) *S*. cf. *convexus* (MIMB 36704, Brittany, France), chaetigers 2–8, left lateral view. (B) *S*. *uschakowi* (MIMB 36677, Sakhalin Is., Sea of Okhotsk), chaetigers 4–7, left lateral view. (C) *S*. cf. *norrisi* (MIMB 3986, Chile), chaetigers 5–11, left lateral view. (D) *S*. *hakaiensis* n. sp. (MIMB 36709, British Columbia, Canada), chaetigers 3–7, left lateral view. (E) *S*. *norrisi* (MIMB 28153, San Diego, California, USA), chaetigers 4–8, left lateral view. Abbreviations: *ch6* –chaetiger 6. Scale bars: A, B– 200 μm; C–E– 100 μm.

*Spiophanes bombyx*: McIntosh [15]: 182–186; [16]: pl XCIII, Fig 1, pl XCVI, Fig 14, pl XCVII, Fig 6, pl XCVIII, Fig 10, pl CV, Fig 9, pl CVI, Fig 9, pl CVII, Fig 16. Söderström [21]: 243–244, Fig 135. Fauvel [140]: 41, Figs 14a–i. Thorson [25]: 91–92, Fig 44 (larval morphology). Hannerz [163]: 33–36, Fig 9 (larval morphology). Hartmann-Schröder [164]: 327–329, Fig 112; [165]: 341–342, Fig 156. Böggemann [32]: 121. Meißner [8] (*Part*.): 54–58, Fig 35. Meißner & Blank [111] (*Part*.): 7–11, Figs 2, 4A, B, D. Delgado-Blas *et al*. [162] (*Part*.): 340.

**Adult morphology** (based on specimens from the Norwegian and North Seas, and around the British Isles and Iceland). McIntosh [15] noted that worms from St Andrews, Scotland, UK, were about 3 inches (76 mm) long for about 180 chaetigers; Meißner & Blank [111] examined specimens 0.2–1.5 mm wide.

Worms examined in the present study 0.3–1.5 mm wide, up to 30 mm long for 120 chaetigers. Pigmentation absent on body and palps. In large formalin-fixed specimens, lateral sides of chaetigers 9–14 (until chaetigers 16–17 in large specimens) light brownish-red due to fixed internal content of large, probably glandular, epithelial cells (Fig 5A and 5B).

Prostomium triangular, wide anteriorly, with a pair of long, distally pointed fronto-lateral horns (Fig 5C), posteriorly narrowed, pressed into chaetiger 1 but not extending over as a caruncle. Peristomium with narrow lateral lips closely applied to lateral sides of prostomium (Fig 5C), and a small ventral lip. Two pairs of small dark red eyes usually present; occasionally eyes absent. Occipital antenna absent. Palps as long as 7–15 chaetigers.

Nuchal organs metameric, at least on 20 anterior chaetigers, fewer in small individuals. First pair of metamers long, oblique ciliary bands extending from posterior part of prostomium to end of chaetiger 2, shorter in small individuals; posterior ends of metamers set wider apart. Succeeding metamers shorter, each extending from nototroch over posterior half of chaetiger, oriented parallel to body axis until chaetigers 13–14, oblique on succeeding chaetigers.

Notochaetae capillaries, long on four anterior chaetigers and on posterior chaetigers, shorter on middle chaetigers. Neuropodia of chaetiger 1 each with 1–2 heavy recurved, crook-like spines in addition to capillaries (Fig 6A). Notopodia of 5–10 posterior chaetigers each with 1–3 (usually one) long recurved spines in addition to capillaries (Fig 6E).

Sabre chaetae in neuropodia from chaetiger 15, one or rarely two in a tuft, with narrow limbation and fine granulation on distal part (Fig 6B–6D).

Hooks in neuropodia from chaetiger 15, up to 10 in a series, accompanied by inferior sabre chaetae throughout body and 1–2 short hair-like alimbate capillaries in 5–10 posterior chaetigers; hair-like capillaries in posterior neuropodia usually situated in upper and/or lower parts of hook row (Fig 6D). Hooks usually quadridentate, with three small upper teeth above main fang and small subterminal hood below main fang (Fig 6B–6D); median upper tooth occasionally weakly developed or double.

Pygidium with small fleshy ventral pad and one pair of thin long lateral cirri (Fig 5D).

Glandular organs in chaetigers 5–14, largest in chaetigers 7 and 8, smallest in chaetiger 9 and gradually increasing in size from chaetiger 10 to chaetigers 12–13, slightly smaller again in chaetiger 14 (Figs 5B and 7A–7F); large organs occupying most of chaetiger space. Internal fibres long and coiled in glandular organs in chaetigers 5–8 (Fig 7B and 7C), straight, shorter and thinner in chaetigers 9–14; in fixed specimens, long straight fibers usually protruding from openings of glandular organs on chaetiger 6. Each organ on chaetigers 5, 7 and 8 opening to exterior via semicircular to suboval slit around large fiber spreader, on chaetiger 6 via small round hole, and on chaetigers 9–14 via large vertical slit. Frontal edge of fiber each spreader on chaetigers 5, 7 and 8 entire, rounded to blunt, or with variously developed middle depression vaguely separating two rounded lobes.

Digestive tract with gizzard-like structure beginning from chaetigers 15–20 and extending through 2–3 chaetigers.

Main dorsal blood vessel with heart body 10–20 μm in diameter.

Nephridia from chaetiger 15 onwards.

*Methyl green staining*. Intensely stained lateral sides of chaetiger 6; weakly stained lateral sides of chaetiger 5. This pattern was observed in small and large specimens from the North and Norwegian seas. In large specimens, the dorsal side of the prostomium and lateral sides of the peristomium were usually also intensely stained.

*Reproduction*. *Spiophanes* cf. *bombyx* is gonochoristic. The gametes proliferate in paired gonads attached to the genital blood vessels from chaetigers 18–19 to chaetigers 100–110. In females, oogenesis is intraovarian: vitellogenesis occurs when the oocytes grow in ovaries. The developed oocytes are accumulated in the coelomic cavity prior to spawning (Fig 8A). The largest intraovarian oocytes examined in fixed specimens from Norway were of irregular shape, up to 105 μm in diameter, each with a honey-combed envelope 3–4 μm thick, 45–55 cortical alveoli regularly arranged in a peripheral circle, a nucleus about 55 μm in diameter, and a single nucleolus about 20 μm in diameter. The cortical alveoli were pear-shaped, up to 8 μm long and 5 μm in diameter, with narrow necks oriented perpendicular to the oocyte surface (Fig 8B–8E).

In males, spermatogonia proliferate in testes and the rest of spermatogenesis occurs in the coelomic cavity. Spermatids are interconnected in tetrads. The spermatozoa are ect-aquasperm with a short plate-like acrosome, subspherical nucleus about 3 μm in diameter, very short midpiece, and a long flagellum (Fig 8F).

Females and males release their gametes into the water where fertilization and holopelagic, planktotrophic larval development occur. Larvae have two pairs of red eyes on the prostomium and a midventral ciliated pit on chaetiger 2 (see Hannerz [163]: Fig 9a, 9b).

*Remarks*. Horned *Spiophanes* from the North and Norwegian seas morphologically appear similar to the individual from the Adriatic Sea and almost fit the original and later descriptions of *S*. *bombyx* from the Mediterranean. All the adults have sabre chaetae and hooks in neuropodia from chaetiger 15 onwards. They differ, however, in the number of cortical alveoli which are regularly arranged in the peripheral circle in mature oocytes. Claparède ([9]: 486, pl 12, Fig 2E, 2F) noted that in worms from the Gulf of Naples the developed oocytes were lentiform (sphéroïdes aplatis), up to 130 μm in diameter, with thick papillary envelope, a large spherical germinal vesicle and a spherical nucleolus; 18 to 20 colorless cortical alveoli (“vésicules inclores” in his terminology), each about 11 μm in diameter, were arranged in a large circle. We observed 45–55 alveoli per oocyte in worms from the Norwegian Sea (Fig 8B–8E). Sequences of the North European and single Italian specimen obtained in the present study were similar (*p*-distances ranging from 00% for *28S* to 2.46% for *16S*). Nevertheless, because of the difference in the number of cortical alveoli in oocytes (which is supposed to be species specific and variable within a small range), and the interrupted distribution of the North European and the Mediterranean populations (see below *S*. cf. *convexus*), we suggest that the conspecificity of these populations should be verified in additional molecular analysis based on sequences of more genes and more individuals. Pending molecular data, we refer to the specimens from the North and Norwegian seas, and also from around Iceland and British Isles as *S*. cf. *bombyx*.

The larvae and juveniles of *S*. cf. *bombyx* were described from Frederikshavn, NW Kattegat, Denmark, by Thorson [25] and from Gullmar Fjord, Sweden, by Hannerz [163]. Hannerz [163] noted that in Gullmar Fjord 18-chaetiger juvenile about 1.3 mm long had characteristic recurved spines in neuropodia of chaetiger 1, and hooks in neuropodia from chaetiger 13.

*Distribution*. Northern Europe (southern limits uncertain) (Fig 4A).

***Spiophanes* cf. *convexus* Delgado-Blas *et al*., 2019.** Figs 9 and 10A.

?*Spiophanes convexus* Delgado-Blas *et al*. 2019 [162]: 344–347, Figs 5, 6.

*Spiophanes bombyx*:? Cazaux [166]: 207–210, pl 68, Figs 1–6. Meißner [8] (*Part*.): 54–58, Figs 33, 34. Meißner & Blank [111] (*Part*.): 7–11. Aylagas *et al*. [121]: S1 Table. Not Claparède [9, 10].

*Type material*. SPAIN, **Galicia**, Pontevedra: N° 9 Meira, 42.2833°N, 8.7167°W, Jun 76, MNCN 16.01/18442 (holotype); N° 10 Meira, 42.2833°N, 8.7167°W, Jun 76, MNCN /16.01/18444 (2 paratypes). **Andalusia**, Huelva, mouth of River Piedras, M.2, 37.2°N, 7.1167°W, 1988, MNCN 16.01/18443 (11 paratypes).

**Adult morphology** (based on our material from Brittany, France). Up to 30 mm long, 1 mm wide for 120 chaetigers. Pigmentation absent on body and palps. In formalin-fixed specimens, lateral sides of chaetigers 9–14 (until chaetigers 16–17 in large specimens) light brownish due to fixed internal content of large, probably glandular, epithelial cells.

Prostomium triangular, wide anteriorly, with a pair of distally pointed fronto-lateral horns, posteriorly narrowed, pressed into chaetiger 1 but not extending over as a caruncle. Fronto-lateral horns long (Fig 9C) or moderately developed (Fig 9D). Peristomium with narrow lateral lips closely applied to lateral sides of prostomium, and a small ventral lip. Two pairs of small dark red eyes present or eyes absent. Occipital antenna absent. Palps as long as 7–15 chaetigers.

Nuchal organs metameric, at least on 40 anterior chaetigers, fewer in small individuals. First pair of metamers long oblique ciliary bands extending from posterior part of prostomium to end of chaetiger 2; posterior ends of metamers set wider apart. Succeeding metamers shorter, each extending from nototroch over posterior half of chaetiger, oriented parallel to body axis until chaetigers 10–11, slightly oblique on succeeding chaetigers. Epithelial cells around nuchal patches with dark yellow pigment in life.

Notochaetae capillaries, long on four anterior chaetigers and on posterior chaetigers, shorter on middle chaetigers. Neuropodia of chaetiger 1 each with 1–2 heavy recurved, crook-like spines in addition to capillaries (Fig 9F). Notopodia of 5–10 posterior chaetigers each with 1–2 (usually one) long recurved spines in addition to capillaries (Fig 9H).

Sabre chaetae in neuropodia from chaetiger 15, one or rarely two in a tuft, with narrow limbation and fine granulation on distal part.

Hooks in neuropodia from chaetiger 15, up to 10 in a series, accompanied by inferior sabre chaetae throughout body and 1–2 short hair-like alimbate capillaries in 5–10 posterior chaetigers; hair-like capillaries in posterior neuropodia usually situated in upper and/or lower parts of hook row. Hooks usually quadridentate, with three small upper teeth above main fang and small subterminal hood below main fang (Fig 9G).

Nototrochs from chaetiger 1 to end of body; on each of chaetigers 1 and 2 as two short, oblique ciliary bands situated between notopodial postchaetal lamellae and nuchal ciliary bands; from chaetiger 3 through most succeeding chaetigers as complete transverse bands of dense cilia running between notopodial postchaetal lamellae. Each nototroch composed of two parallel rows of large transversally elongated cells, each bearing numerous long cilia; nototroch ciliation stronger on ridge-bearing chaetigers. Single transverse rows of smaller cells with shorter cilia present on each chaetiger beginning from chaetiger 3.

Dorsal transverse ridges present from chaetigers 14–15 to chaetigers 40–50, fewer in small individuals. Ridges thick, fleshy, one per chaetiger, not connected to notopodial lamellae. Wide blood vessel extending along each ridge internally; double nototroch with numerous long cilia arranged on top of each ridge. Intersegmental lateral pouches absent.

Pygidium with small fleshy ventral pad and one pair of thin long lateral cirri (Fig 9H and 9I).

Glandular organs in chaetigers 5–14, largest in chaetigers 7 and 8, smallest in chaetiger 9 and gradually increasing in size from chaetiger 10 to chaetigers 12–13, slightly smaller again in chaetiger 14; large organs occupying most of chaetiger space. Internal fibres long and coiled in glandular organs in chaetigers 5–8, straight, shorter and thinner in chaetigers 9–14; in fixed specimens, long straight fibers usually protruding from openings of glandular organs on chaetiger 6 (Fig 9E). Each organ on chaetigers 5, 7 and 8 opening to exterior via semicircular to suboval slit around large fiber spreader, on chaetiger 6 via small round hole, and on chaetigers 9–14 via large vertical slit (Fig 9A, 9B and 9E). Frontal edge of each fiber spreader on chaetigers 5, 7 and 8 entire, rounded to blunt, or with variously developed middle depression vaguely separating two rounded lobes.

Digestive tract with gizzard-like structure beginning from chaetigers 15–20 and extending through 2–3 chaetigers.

Main dorsal blood vessel with heart body 10–20 μm in diameter.

Nephridia from chaetiger 15 onwards.

*Methyl green staining*. Intensely stained lateral sides of chaetiger 6; weakly stained lateral sides of chaetigers 8–14; no staining on head or chaetigers 1–5, 7 (Figs 9A, 9B and 10A).

*Remarks*. Horned *Spiophanes* from Brittany, northern France, morphologically appear very similar or almost identical to *S*. *bombyx* from the Mediterranean and *S*. cf. *bombyx* from the North and Norwegian seas, but unambiguously differ from them in genetic characteristics (Figs 1B and 2B). They weakly differ from North European specimens also by the pattern of MG staining: no staining on head and five anterior chaetigers in *S*. cf. *convexus*, and staining on chaetiger 5 and often on the prostomium and peristomium in *S*. cf. *bombyx*. However, these patterns are variable and can only be seen in well preserved specimens.

Delgado-Blas *et al*. [162] described *S*. *convexus* based on the morphology of a few anterior fragments from Atlantic Spain (Galicia and Andalusia), and also from Mediterranean Spain (Catalonia). The diagnostic characters used to distinguished *S*. *convexus* from *S*. *bombyx* were ambiguous and fit into the range of morphological variability of *S*. *bombyx*, which was not examined by Delgado-Blas *et al*. [162]. Based on the proximity of the type locality of *S*. *convexus* (Meira, Pontevedra, Galicia) and sampling sites of specimens examined through molecular analysis in the present study, we assume that our specimens from Brittany, France, and specimens from Bay of Biscay sequenced by Aylagas *et al*. [121] belong to *S*. *convexus*. In the absence of sequence data of *S*. *convexus*, however, we refer our specimens to as *S*. cf. *convexus*. The identity of the horned *Spiophanes* from Catalonia, Spain, referred by Delgado-Blas *et al*. [162] to as *S*. *convexus*, should be verified in molecular analysis. So far, based on available data, we consider *S*. *bombyx* as the only horned species occurring in the Mediterranean.

To avoid confusion in identification of specimens based on morphological characters only, especially in areas of possible overlap of *S*. *bombyx* and *S*. *convexus*, and until more molecular data has been obtained from specimens from the Gulf of Naples, we suggest that North European horned *Spiophanes* are referred to as *S*. *bombyx* aggregate (*S*. *bombyx* agg.), rather than to a particular species.

Mesnil [11] described in detail adults and larvae of *S*. *bombyx* from Wimereux, Hauts-de-France, northern France. He noted that the adults were up to 6 cm long, 1.5 mm wide for about 180 chaetigers. They had hooks beginning invariably from chaetiger 15, whereas in larvae hooks started from chaetigers 11–14. Wimereux is located midway between the sites where *S*. cf. *bombyx* and *S*. cf. *convexus* were collected for molecular analysis. Because Mesnil’s [11] description fits both species, the identity of the worms described by him from Wimereux remains uncertain.

Cazaux [166] described in detail the larval development of a horned *Spiophanes* from Arcachon, France, which he identified as *S*. *bombyx*. He noted that in an early benthic 15-chaetiger juvenile, hooks were not yet developed, and in a 19-chaetiger juvenile they were present in chaetigers 13–19. Arcachon is located between sites where specimens of *S*. cf. *convexus* were collected for molecular analysis. It is plausible, therefore, that Cazaux [166] dealt with *S*. *convexus* but not *S*. *bombyx*.

*Distribution*. Western Europe (northern and southern limits uncertain) (Fig 4A).

**Fig 11:**
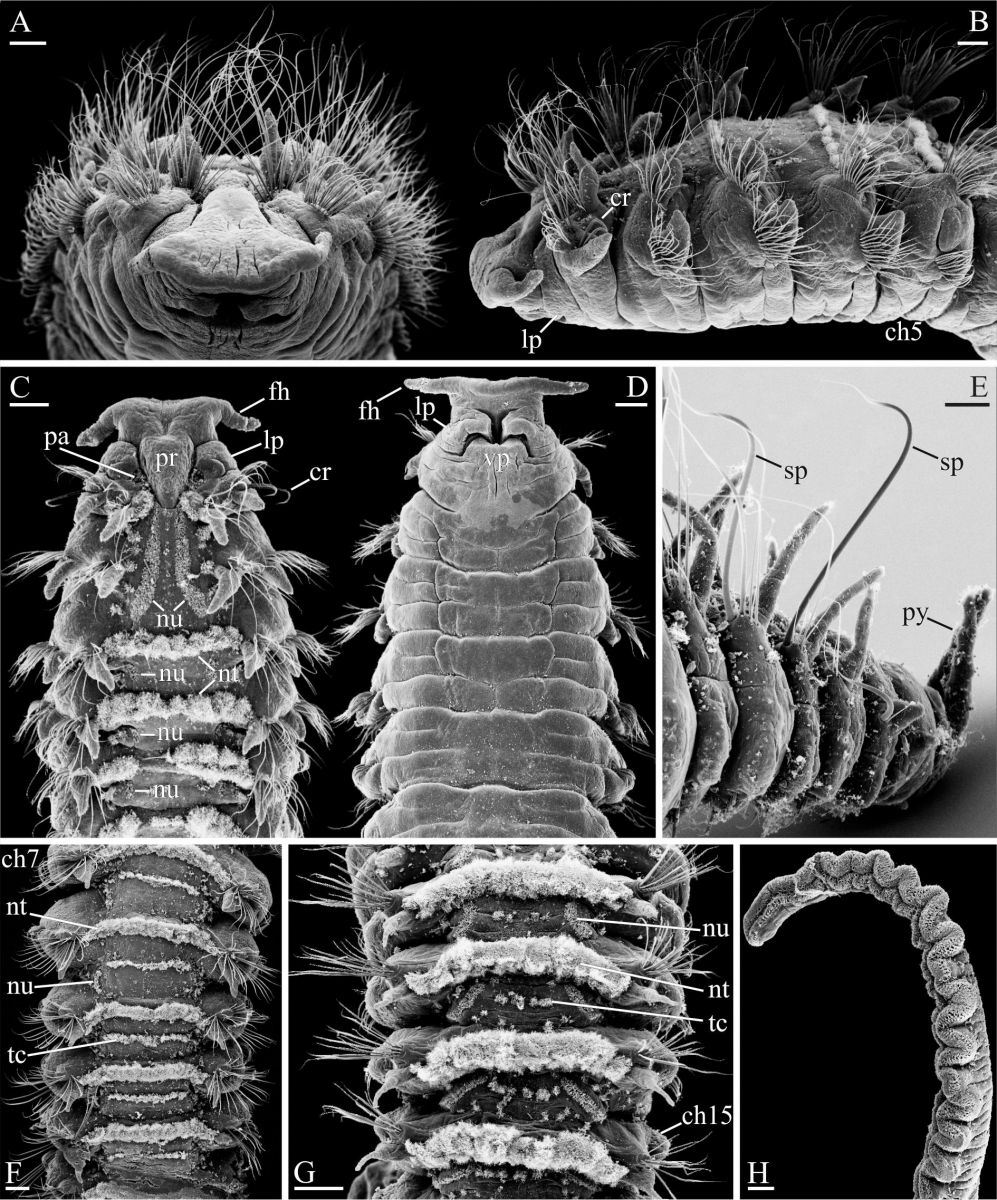
Adult morphology of *Spiophanes uschakowi* (Peter the Great Bay, Russia, USNM 183505). (A) Anterior end, frontal view. (B) Same, left lateral view. (C) Same, dorsal view, palps missing. (D) Same, ventral view. (E) Posterior end, left lateral view. (F) Chaetigers 7–11, dorsal view, showing longitudinal nuchal metamers, nototrochs and intersegmental transverse bands of cilia. (G) Chaetigers 12–15, dorsal view, showing oblique nuchal metamers, nototrochs and intersegmental transverse bands of cilia. (H) Palp, fronto-lateral view, showing frontal longitudinal ciliated groove. Abbreviations: *ch5*, *ch7*, *ch15* –chaetigers 5, 7, 15; *cr*–crook-like spine in neuropodium of chaetiger 1; *fh*–fronto-lateral horn; *lp*–lateral peristomial lip; *nt*–nototroch; *nu*–nuchal organ; *pa*–palp scar; *pr*–prostomium; *py*–pygidial cirrus; *sp*–notopodial spine with recurved distal end; *tc*–intersegmental transverse cilia; *vp*–ventral peristomial lip. Scale bars: A– 50 μm; B–H– 100 μm.

**Fig 12:**
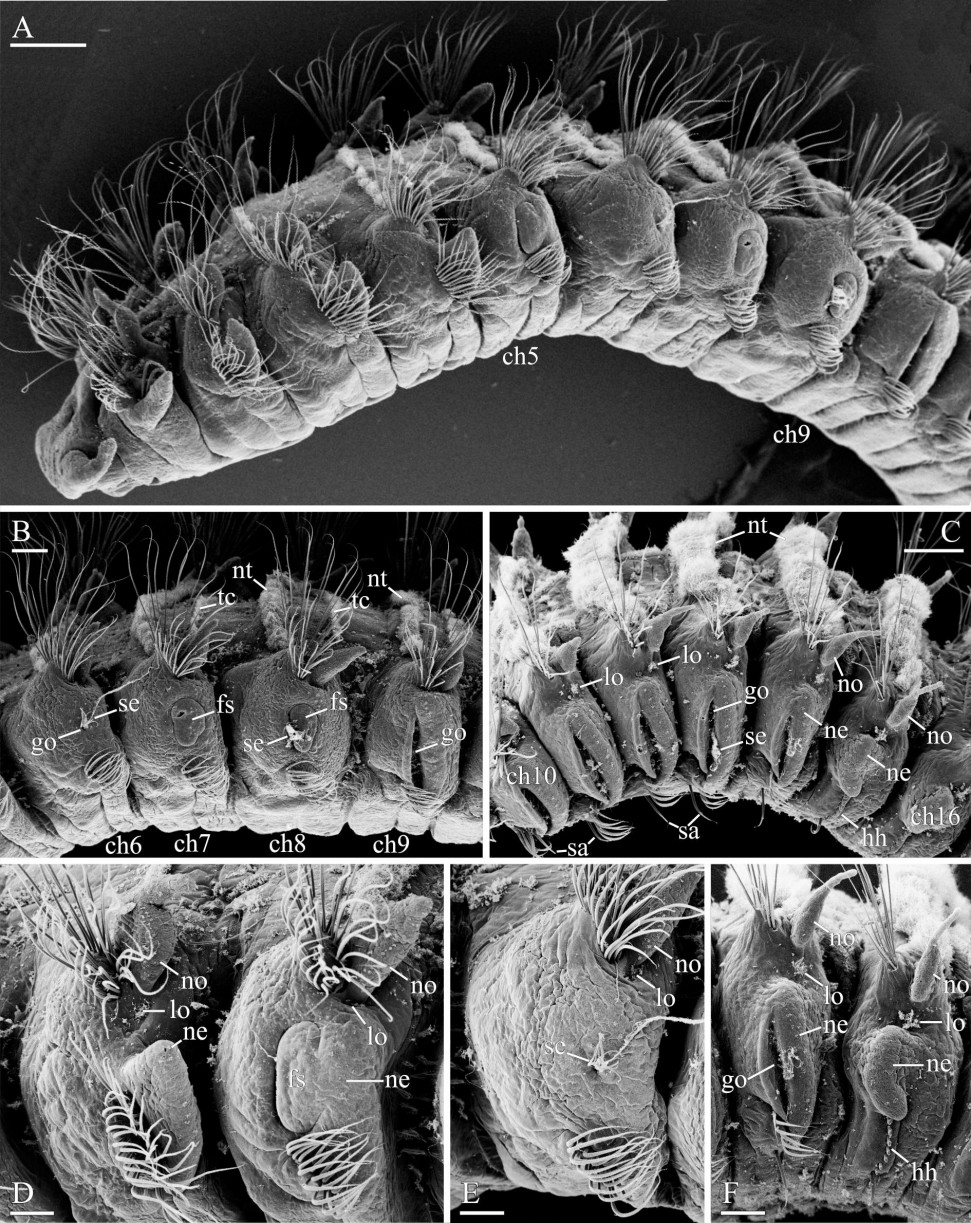
Glandular organs of *Spiophanes uschakowi* (Peter the Great Bay, Russia, USNM 183505). (A) Anterior end, left lateral view. (B) Chaetigers 6–9, left lateral view, showing openings of glandular organs: small hole on chaetiger 6, suboval slits on chaetigers 7 and 8, and vertical slit on chaetiger 9. (C) Chaetigers 10–16, left lateral view, showing slit-like openings of glandular organs on chaetigers 10–14. (D) Chaetigers 4 and 5, left lateral view, showing suboval fiber spreader on neuropodium of chaetiger 5. (E) Chaetiger 6, left lateral view, showing hole-like opening of glandular organ in neuropodium. (F) Chaetigers 14 and 15, left lateral view, showing slit-like opening of glandular organ in neuropodium of chaetiger 14 and hooks in neuropodia of chaetiger 15. Abbreviations: *ch5*–*ch16* –chaetigers 5–16; *fs*–fiber spreader; *go*–opening of glandular organ; *hh*–hooded hook; *lo*–lateral sensory ciliated organ; *ne*–neuropodial postchaetal lamella; *no*–notopodial postchaetal lamella; *nt*–nototroch; *sa*–ventral inferior sabre chaeta; *se*–hardened secretion protruding from opening of glandular organ; *tc*–intersegmental transverse cilia. Scale bars: A– 200 μm; B, C– 100 μm; D–F– 50 μm.

**Fig 13:**
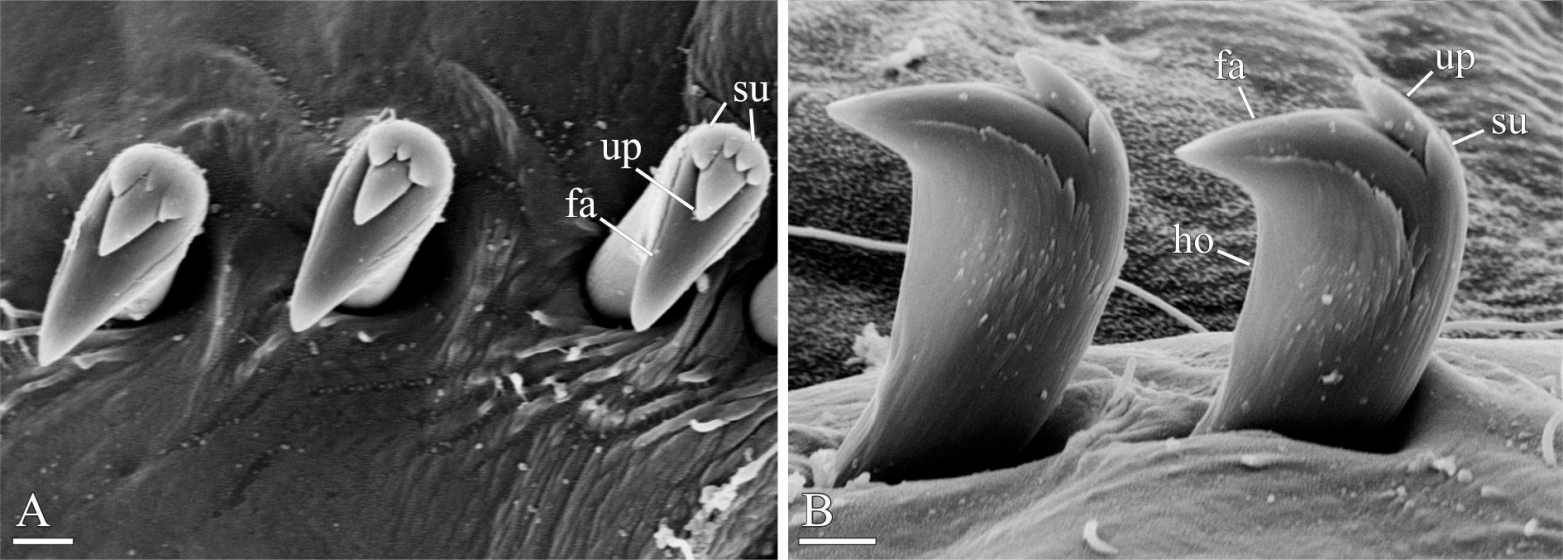
Adult morphology of *Spiophanes uschakowi* (Peter the Great Bay, Russia, USNM 183505). (A) Hooks in neuropodium of chaetiger 15, view from above, showing variation in number of superior teeth. (B) Same, lateral view, showing small subterminal hood below main fang. Abbreviations: *fa*–main fang of hook; *ho*–subdistal hood below main fang of hook; *su*–superior teeth of hook; *up*–upper tooth of hook. Scale bars: A, B– 2 μm.

**Fig 14:**
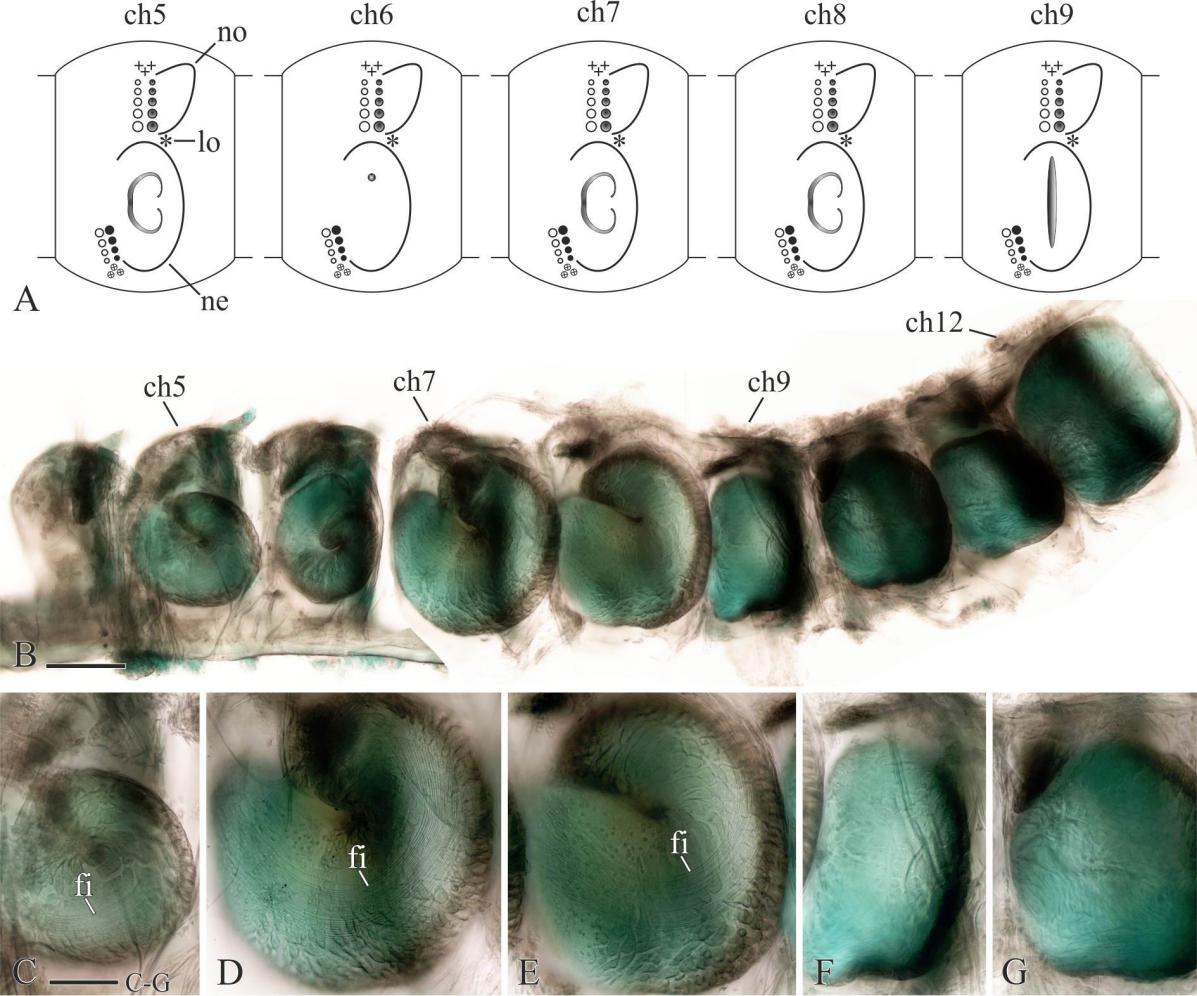
Glandular organs of *Spiophanes uschakowi* (Sakhalin Is., Sea of Okhotsk, MIMB 36677). (A) Schematic composition of parapodia and shape of openings of glandular organs on neuropodia of chaetigers 5–9: suboval openings with one-lobe chaetal spreader on chaetigers 5, 7 and 8; small hole on chaetiger 6; and a vertical slit on chaetiger 9. (B–G) Morphology of glandular organs in chaetigers 5–12, right-side organs in left lateral view, formalin-fixed specimen stained with an alcohol solution of methyl green. (B) Chaetigers 5–12. (C) Chaetiger 5. (D) Chaetiger 7. (E) Chaetiger 8. (F) Chaetiger 9. (G) Chaetiger 10. Abbreviations: *ch5*–*ch12* –chaetigers 5–12; *fi*–fibers produced by glandular organs; *lo*–lateral sensory ciliated organ; *ne*–neuropodial postchaetal lamella; *no*–notopodial postchaetal lamella. Scale bars: B– 200 μm; C–G– 100 μm.

***Spiophanes uschakowi* Zachs, 1933.** Figs 10B and 11–14

*Spiophanes uschakowi* Zachs, 1933 [112]: 130. Meißner [8]: 61. Meißner & Blank [111]: 15–17, Fig 7. Buzhinskaja [84]: 61. Radashevsky *et al*. [167]: 33–39, Figs 1–6 (gamete ultrastructure).

*Spiophanes uschakovi* [sic]: Annenkova [168]: 140; [113]: 172–173. Uschakov [78]: 267.

*Spiophanes bombyx*: Okuda [76]: 222–223, Figs 3, 4. Annenkova [113]: 172. Uschakov [77]: 200; [78]: 267; [169]: 205. Pasternak [170]: 267. Imajima & Hartman [80]: 289–290. Buzhinskaja [114]: 103; [83]: 135; [84]: 61. Koblikov [171]: 39. Ozolinsh [172]: 82–83. Imajima [88]: 128–132, Figs 8, 9; [116]: 129; [117]: 80; [118]: 379. Buzhinskaja & Britayev [115]: 85. Ozolinsh & Bagaveeva [173]: 139. Not Claparède [9, 10].

*Spiophanes* sp.: Radashevsky [174]: Figs 1F, 5D, 7B, 10D.

*Type material*. RUSSIA, **Primorsky Region**, Sea of Japan (East Sea), Peter the Great Bay, 42.835°N, 131.74°E, dredge, 26–30 m, fine sand, coll. K.M. Derjugin, 11 Oct 1925, ZISP 1/25826 (2 syntypes).

**Adult morphology**. Up to 47 mm long, 1.5 mm wide for 140 chaetigers. Pigmentation absent on body and palps. A pair of narrow transverse whitish bands, concentrations of epithelial glandular cells, visible on ventral side of chaetiger 1 in live individuals. In large formalin-fixed specimens, lateral sides of chaetigers 9–14 (until chaetigers 16–17 in large specimens) light brownish-red due to fixed internal content of large, probably glandular, epithelial cells.

Prostomium triangular, wide anteriorly, with a pair of long, distally pointed fronto-lateral horns (Fig 11A–11D), posteriorly narrowed, pressed into chaetiger 1 but not extending over it as a caruncle. Peristomium with narrow lateral lips closely applied to lateral sides of prostomium, and a small ventral lip (Fig 11B–11D). Two pairs of small dark red eyes arranged trapezoidally, comprising one pair of median eyes and one pair of lateral eyes situated anteriorly and set wider apart; occasionally eyes absent. Occipital antenna absent. Palps as long as 7–15 chaetigers, with deep frontal longitudinal groove lined with fine cilia, fronto-lateral motile compound cilia bordering frontal groove, short transverse rows of short motile compound cilia regularly arranged on inner lateral side and beating towards frontal groove, short compound non-motile cilia arising directly from palp surface and scattered on lateral and abfrontal palp surfaces (Fig 11H).

Nuchal organs metameric, at least on 25 anterior chaetigers, fewer in small individuals. First pair of metamers long oblique ciliary bands extending from posterior part of prostomium to end of chaetiger 2, shorter in small individuals; posterior ends of metamers set wider apart (Fig 11C). Succeeding metamers shorter, each extending from nototroch over posterior half of chaetiger, oriented parallel to body axis until chaetigers 9–10 (Fig 11F), oblique on succeeding chaetigers (Fig 11G).

Notochaetae capillaries, long on four anterior chaetigers and on posterior chaetigers, shorter on middle chaetigers. Neuropodia of chaetiger 1 each with 1–2 heavy recurved, crook-like spines in addition to capillaries (Figs 11A–11C and 12A). Notopodia of 5–10 posterior chaetigers each with 1–3 (usually one) long spines with recurved distal end in addition to capillaries (Fig 11E). Noto- and neuropodial postchaetal lamellae on four anterior chaetigers subtriangular, with pointed tips (Figs 11A–11D and 12A); notopodial lamellae on succeeding middle chaetigers short, subulate (Fig 11F and 11G), on posterior chaetigers long, cirriform (Fig 11E). Neuropodial postchaetal lamellae on chaetigers 5–14 large but low, rounded and fleshy, with openings of internal glandular organs (Figs 11B and 12A–12F).

Sabre chaetae in neuropodia from chaetiger 10, usually one, occasionally two per neuropodium, with fine granulation on distal part; chaetae on chaetiger 10 comparatively small, with narrow limbation, slightly reducing in size until chaetiger 14, but from chaetiger 15 onwards thicker, alimbate (Fig 12C).

Hooks in neuropodia from chaetiger 15, up to 15 in a series, accompanied by inferior sabre chaetae throughout body and 1–2 hair-like alimbate capillaries in 7–10 posterior chaetigers; hair-like capillaries in posterior neuropodia usually situated in upper and/or lower parts of hook row. Hooks usually quadridentate, with three small upper teeth above main fang and small subterminal hood below main fang (Fig 13B); median upper tooth occasionally weakly developed or doubled (Fig 13A and 13B).

Nototrochs from chaetiger 1 to end of body; on each of chaetigers 1 and 2 as two short, oblique ciliary bands situated between notopodial postchaetal lamellae and nuchal ciliary bands; from chaetiger 3 onwards as complete transverse bands of dense cilia running between notopodial postchaetal lamellae (Figs 11C, 11F, 11G and 12C). Each nototroch composed of two parallel rows of large transversally elongated cells, each bearing numerous long cilia; nototroch ciliation stronger on ridge-bearing chaetigers. Single transverse rows of smaller cells with shorter cilia present on each chaetiger beginning from chaetiger 3. These rows situated posterior to nototrochs and referred to as intersegmental transverse ciliation (Fig 11F and 11G).

Dorsal transverse ridges present from chaetiger 15 to chaetigers 30–55, fewer in small individuals. Ridges thick, fleshy, one per chaetiger, not connected to notopodial lamellae. Wide blood vessel extending along each ridge internally; double nototroch with numerous long cilia arranged on top of each ridge. Intersegmental lateral pouches absent.

Pygidium with small fleshy ventral pad and one pair of thin long lateral cirri (Fig 11E); one to two additional cirri present above lateral cirri in some individuals.

Glandular organs in chaetigers 5–14, largest in chaetigers 7 and 8, smallest in chaetiger 9 and gradually increasing in size from chaetiger 10 to chaetigers 12–13 (Fig 14B–14G), slightly smaller again in chaetiger 14; large organs occupying most of chaetiger space. Internal fibres long and coiled in glandular organs in chaetigers 5–8 (Fig 14B–14E), straight, shorter and thinner in chaetigers 9–14; in fixed specimens, long straight fibers usually protruding from openings of glandular organs on chaetiger 6. Each organ on chaetigers 5, 7 and 8 opening to exterior via semicircular to suboval slit around large fiber spreader (Figs 11B, 12A, 12B, 12D and 14A), on chaetiger 6 via small round hole (Figs 12E and 14A), and on chaetigers 9–14 via large vertical slit (Figs 12A–12C, 12F and 14A). Frontal edge of each fiber spreader on chaetigers 5, 7 and 8 entire, rounded to blunt, or with variously developed middle depression vaguely separating two rounded lobes (Fig 12A, 12B and 12D).

Foregut wide, eversible, within 3–6 anterior chaetigers, with black pigment in thick wall; oesophagus in succeeding chaetigers narrow, with thin unpigmented wall. Gizzard-like structure in end of oesophagus beginning from chaetigers 14–21 and extending through 2–3 chaetigers.

Main dorsal blood vessel with heart body extending from chaetigers 14–16 to chaetigers 20–21. Heart body soft, brownish-green, 30–40 μm in diameter. Main dorsal blood vessel transforming into gut sinus 1–3 chaetigers after gizzard-like structure. Circumoesophageal vessels joined midventrally in chaetiger 3 forming main ventral blood vessel. Blood red, without elements.

Nephridia from chaetiger 15 onwards, opening to exterior on antero-lateral edges of chaetigers in both sexes.

*Habitat*. Adults of *S*. *uschakowi* live in tubes in sandy to muddy-sand sediments in shallow waters. Population densities reach several thousand individuals per square meter. The tubes are sandy, firm, with thin but strong inner lining, up to 10 cm long and 2 mm in diameter, oriented vertically in sediment, with upper part 2–5 mm long protruding above the surface.

*Methyl green staining*. Intensely stained small area on lateral sides of chaetiger 5 (in front of openings of glandular organs; not stained in some individuals), larger area on chaetiger 6 (Fig 10B) and small fleshy pygidium (pygidial cirri not stained); weakly stained lateral sides of chaetigers 9–14.

*Reproduction*. *Spiophanes uschakowi* is gonochoristic. The gametes proliferate in paired gonads attached to the genital blood vessels from chaetigers 20–24 to chaetigers 88–120. In females, oogenesis is intraovarian: vitellogenesis occurs when the oocytes grow in ovaries. The developed oocytes are accumulated in the coelomic cavity prior to spawning. The newly released oocytes are lentiform, each 185–200 μm in diameter, with honeycombed envelope 5–7 μm thick, 41–49 cortical alveoli regularly arranged in a peripheral circle, a nucleus 80–83 μm in diameter, and a single nucleolus about 30 μm in diameter. The cortical alveoli are pear-shaped, 7–9 μm in diameter, with narrow necks oriented perpendicular to the oocyte surface (see Radashevsky *et al*. [167]: Fig 2).

In males, spermatogonia proliferate in testes and the rest of spermatogenesis occurs in the coelomic cavity. Spermatids are interconnected in tetrads. The spermatozoa are ect-aquasperm with a plate-like acrosome 0.58 ± 0.06 μm thick and 2.14 ± 0.13 μm in diameter, barrel-shaped nucleus 2.23 ± 0.13 μm long and 3.18 ± 0.13 μm in diameter, short midpiece 0.93 ± 0.09 μm long with five spherical mitochondria, two centrioles and one small lipid droplet, and a flagellum 62–63 μm long with 9 × 2 + 2 organization of microtubules (see Radashevsky *et al*. [167]: Fig 5).

Females and males release their gametes into the water where fertilization and holopelagic, planktotrophic larval development occur. Larvae have two pairs of red eyes on the prostomium and a midventral ciliated pit on chaetiger 2. Newly settled 20–21-chaetiger juveniles about 2 mm long had heavy recurved crook-like spines in neuropodia of chaetiger 1, sabre chaetae in neuropodia from chaetiger 10, hooded hooks in neuropodia from chaetiger 14, and long spines with recurved distal end in notopodia from chaetigers 17–19.

*Remarks*. In the original description of *S*. *uschakowi* Zachs ([112]: 130) noticed characteristic feature of worms as notopodial lamellae (“cirri dorsales”) being leaf-like on eight anterior chaetigers and cirriform on the following chaetigers. He noted that hooks appeared rather similar to those in *S*. *bombyx* and incorrectly reported that the first chaetiger had no heavy spines. Annenkova [168, 113] and Uschakov [78] likely had not seen Zachs’ material and distinguished *S*. *bombyx* and *S*. *uschakowi* only according to Zachs’ description. We have examined the two syntypes of *S*. *uschakowi* (two anterior fragments, each about 25–35 chaetigers) deposited in the polychaete collection of the Zoological Institute, Saint Petersburg, Russia (ZISP 1/25826), and observed the heavy recurved spines in first neuropodia, and other characters as they described above.

The ultrastructure of the oocytes and spermatozoa was described in details by Radashevsky *et al*. [167]. The larvae of the species were found in plankton collected in Peter the Great Bay, Sea of Japan (East Sea), Russia, in September-October. Their morphology will be described elsewhere.

*Spiophanes uschakowi* has been recorded from the Shantar Islands, northern part of Sakhalin Is. and from the South Kurile Islands south to the Korean Peninsula. Numerous records of *S*. *bombyx* from around Japanese Islands by Minoru Imajima are herein referred to *S*. *uschakowi* but the conspecificity of mainland and Japanese specimens should be verified in a further study. Remarkably, *S*. *uschakowi* has never been reported from the Chukchi Peninsula, Kamchatka Peninsula, Bering Islands, Kurile Islands, and northern part of the Sea of Okhotsk, although numerous samples from this region were provided by the expeditions of the Institute of Marine Biology, Vladivostok, FEB RAS, the Zoological Institute, St. Petersburg, RAS, some other institutions, and also during this study.

**Fig 15:**
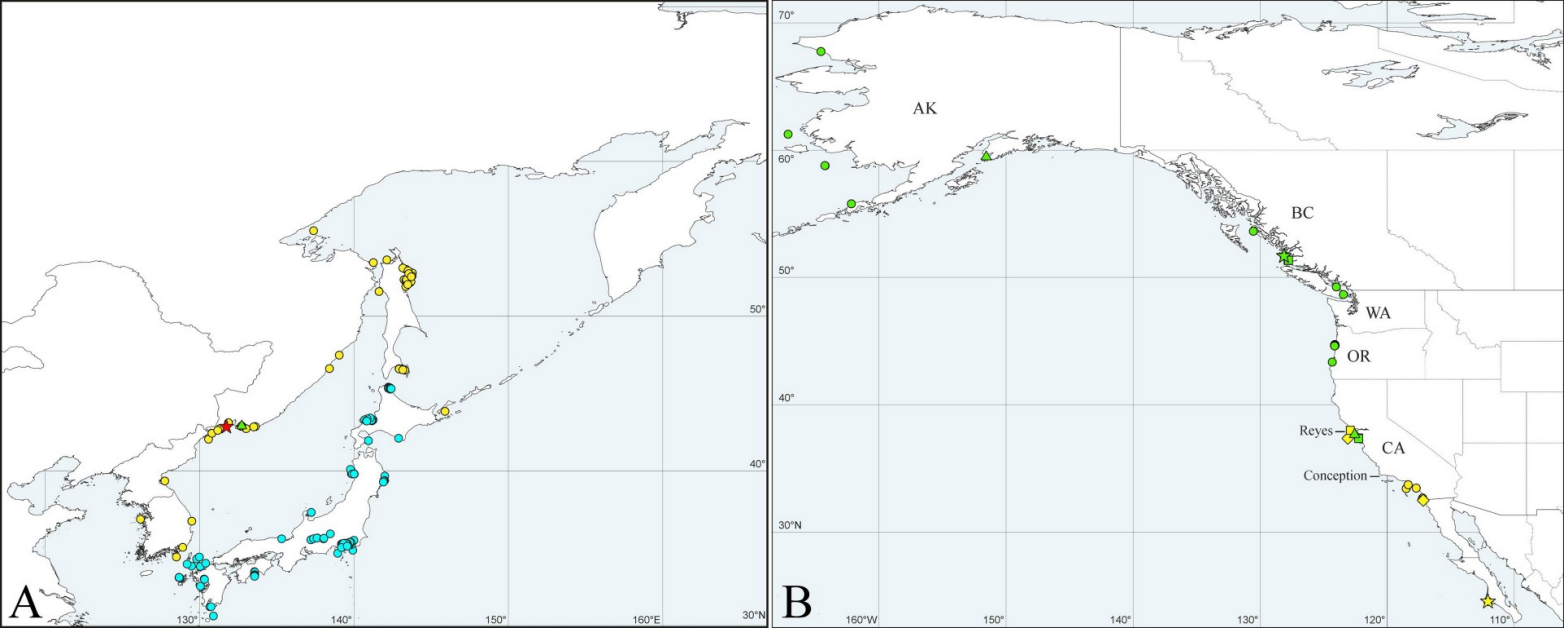
Maps showing records of *Spiophanes* spp. in North Pacific Ocean. (A) ***Spiophanes uschakowi*** Zachs, 1933: red star–type locality: Peter the Great Bay, Sea of Japan (East Sea), Russia; green triangle–specimens sequenced in the present study; yellow circles–adults identified in the present study based on the morphology only; turquoise circles–adults identified as *S*. *bombyx* by Imajima [88, 116–118]. (B) ***Spiophanes norrisi*** Meißner & Blank, 2009 (yellow symbols): yellow star–type locality: Magdalena Bay, Baja California Sur, Mexico; yellow square–specimens sequenced by Meißner & Blank [111]; yellow rhombs–specimens sequenced by the LACM DISCO Project (Los Angeles, California, USA), and the Marine Biology Laboratory (City of San Diego, California, USA); yellow circles–adults identified based on the morphology only. ***Spiophanes hakaiensis*** n. sp. (green symbols): green star–type locality: Calvert Is., British Columbia, Canada; green squares–specimens sequenced in the present study; green triangles–specimens sequenced by the Canadian Centre for DNA Barcoding (Guelph, Canada), and the Marine Biology Laboratory (City of San Francisco, California, USA); green circles–adults identified based on the morphology only.

*Distribution*. North West Pacific: from the Sea of Okhotsk south to the Yellow Sea and East China Sea (Fig 15A).

***Spiophanes norrisi* Meißner & Blank, 2009.** Fig 10E

*Spiophanes norrisi* Meißner & Blank, 2009 [111] (*Part*.): 11–15, Figs 5, 6A, 6B, 6D–6F.

*Spiophanes bombyx*: Hernández-Alcántara *et al*. [63]: 570. Hernández-Alcántara & Solís-Weiss [175]: 29. Méndez [176]: 142. Díaz-Castañeda & Valenzuela-Solano [177]: 513. Not Claparède [9, 10].

*Type material*. MEXICO, **Baja California Sur**, Magdalena Bay, Entrada Point, R/V *Velero IV*, st. 1962–50, 24.545°N, 112.06805°W, rocky intertidal with surfgrass & tide pools, coll. Allan Hancock Foundation, 3 May 1950, LACM-AHF Poly 2251 (holotype), 2254 (16 paratypes).

*Synopsis*. Up to 15 mm long, 0.6 mm wide for 100 chaetigers. Prostomium with long fronto-lateral horns, posteriorly pressed into chaetiger 1 but not extending over it as caruncle. Occipital antenna absent. Nuchal organs metameric, at least on 25 anterior chaetigers; first pair of metamers as oblique ciliary bands from prostomium to end of chaetiger 2; succeeding metamers shorter, parallel to body axis until chaetigers 10–11, oblique on succeeding chaetigers. Sabre chaetae in neuropodia from chaetiger 10. Hooks in neuropodia from chaetiger 15, usually quadridentate, with small subterminal hood. Pygidium with small ventral fleshy pad and one pair of lateral cirri. Glandular organs in chaetigers 5–14, largest in chaetigers 7 and 8, smallest in chaetiger 9, gradually increasing in size from chaetiger 10 to chaetigers 12–13, slightly smaller again in chaetiger 14. Internal fibres long and coiled in glandular organs in chaetigers 5–8, straight, shorter and thinner in chaetigers 9–14. Organs on chaetigers 5, 7 and 8 each opening to exterior via semicircular to suboval slit around large fiber spreader, on chaetiger 6 via small round hole, on chaetigers 9–14 via large vertical slit. Frontal edge of each fiber spreader on chaetigers 5, 7 and 8 entire, rounded to blunt, or with variously developed middle depression vaguely separating two rounded lobes. Digestive tract with gizzard-like structure. Main dorsal blood vessel with heart body. Nephridia from chaetiger 15 onwards. Gonochoristic. Oocytes lentiform, each with thick honey-combed envelope and cortical alveoli regularly arranged in a peripheral circle. Spermatids interconnected in tetrads. Spermatozoa short-headed aqua-sperm with plate-like acrosomes. Fertilization in sea water. Larval development holopelagic, planktotrophic. Larvae with two pairs of red eyes on prostomium and a midventral ciliated pit on chaetiger 2.

*Remarks*. Meißner & Blank [111] designated 17 specimens from one sample collected from Magdalena Bay, Baja California Sur, Pacific Mexico, as the type material of *S*. *norrisi* (Fig 15B). They referred to numerous specimens of horned *Spiophanes* collected along the Pacific American coast from Alaska south to Chile as non-type material of the same species. For molecular analysis (short fragments of *COI*), the authors used specimens collected from offshore San Francisco (SF), northern California.

Molecular analysis performed in the present study suggested the presence of two sibling vicariant species distributed along the Pacific coast of North America and overlapping in California (Figs 2B and 3). Herein, we refer northern specimens to a new species: *Spiophanes hakaiensis* n. sp. (see below). The *COI* sequences provided by Meißner & Blank [111] were grouped together with sequences from specimens from off San Francisco and Baja California Norte, Mexico, provided in other studies (see Table 1, Fig 2B). Consequently, these specimens are referred to as *S*. *norrisi*. South American populations of horned *Spiophanes* have not been examined in genetic analysis. Pending molecular data, we suggest that Chilean and Argentinean specimens are referred to as *S*. cf. *norrisi* (see below).

Meißner & Blank [111] noticed that *S*. *norrisi* differed from morphologically similar species mainly in the orientation of segmental nuchal metamers (“dorsal ciliated patches”, “metameric ciliated patches” or “dorsal ciliated organs” in their terminology), and that it was the only known species with oblique orientation of the metamers from as early as between chaetigers 9–10. After describing horned American *Spiophanes* as a new species, *S*. *norrisi*, Meißner & Blank ([111]: 6) noted that *S*. *uschakowi* “turned out” to be very similar to *S*. *norrisi*, but “a definite assignment [of *S*. *uschakowi*] based on morphological characters was not possible as long as the nature of the dorsal ciliated organs posterior to chaetiger 2 was unknown.” We observed segmental nuchal metamers oriented parallel to body axis until chaetigers 9–10 in *S*. *uschakowi* and until chaetigers 10–11 in *S*. *norrisi*; thus the two species cannot be distinguished based on the morphology of their nuchal organs. We did not observe sabre chaetae beginning earlier than chaetiger 10 in *S*. *norrisi* and interpret their rare start from chaetiger 9 reported by Meißner & Blank [111] as an abnormality, as with the occasional bifurcation of one or both pygidial cirri rarely occurring both in *S*. *uschakowi* and *S*. *norrisi*. Individuals of both species demonstrate the same variability in the shape of the chaetal spreaders on chaetigers 5, 7 and 8, and all other morphological characters examined so far. Specimens of the two species differ slightly in the patterns of the MG staining. In *S*. *uschakowi*, the lateral sides of chaetiger 5 (in front of openings of glandular organs) are usually weakly stained (Fig 10B), whereas in *S*. *norrisi* such staining has not been observed (Fig 10E). The staining pattern in *S*. *uschakowi*, however, is variable and in some individuals (worms from Peter the Great Bay were mostly examined on this account) staining was not revealed on chaetiger 5, making this character also unreliable for distinguishing the two species.

Blake [178] described 13- and 14-chaetiger horned larvae of a *Spiophanes* collected in Tomales Bay, California, in November 1971. The 14-chaetiger larva was 1025 μm long and had hooded hooks in neuropodia from chaetiger 11. Blake [178] noticed subtle morphological differences between the Californian and Swedish larvae (described by Hannerz [163]) and referred the former to as *S*. cf. *bombyx*. However, two other horned *Spiophanes*, *S*. *anoculata* Hartman, 1960 and *S*. *hakaiensis* n. sp., also occur in Californian waters. As with *S*. *bombyx* and *S*. *norrisi*, adults of *S*. *anoculata* have hooded hooks in neuropodia from chaetiger 15. So far, *S*. *anoculata* has only been found in deep water, from 922 m to 2800 m depth (see Meißner [8]), but because Blake [178] collected very few (only two) larvae of same kind in shallow-water plankton, these larvae may belong to a deep-water species. Thus, their identity remains uncertain.

*Distribution*. North East Pacific, California: from San Francisco Bay, USA, south to Baja California Sur, Mexico (Fig 15B).

***Spiophanes hakaiensis* Radashevsky & Pankova, n. sp..** *urn*:*lsid*:*zoobank*.*org*:*act*:20552AB1-C5D1-4310-A8B2-5BCE4370ECFA

Figs 10D and 16

**Fig 16:**
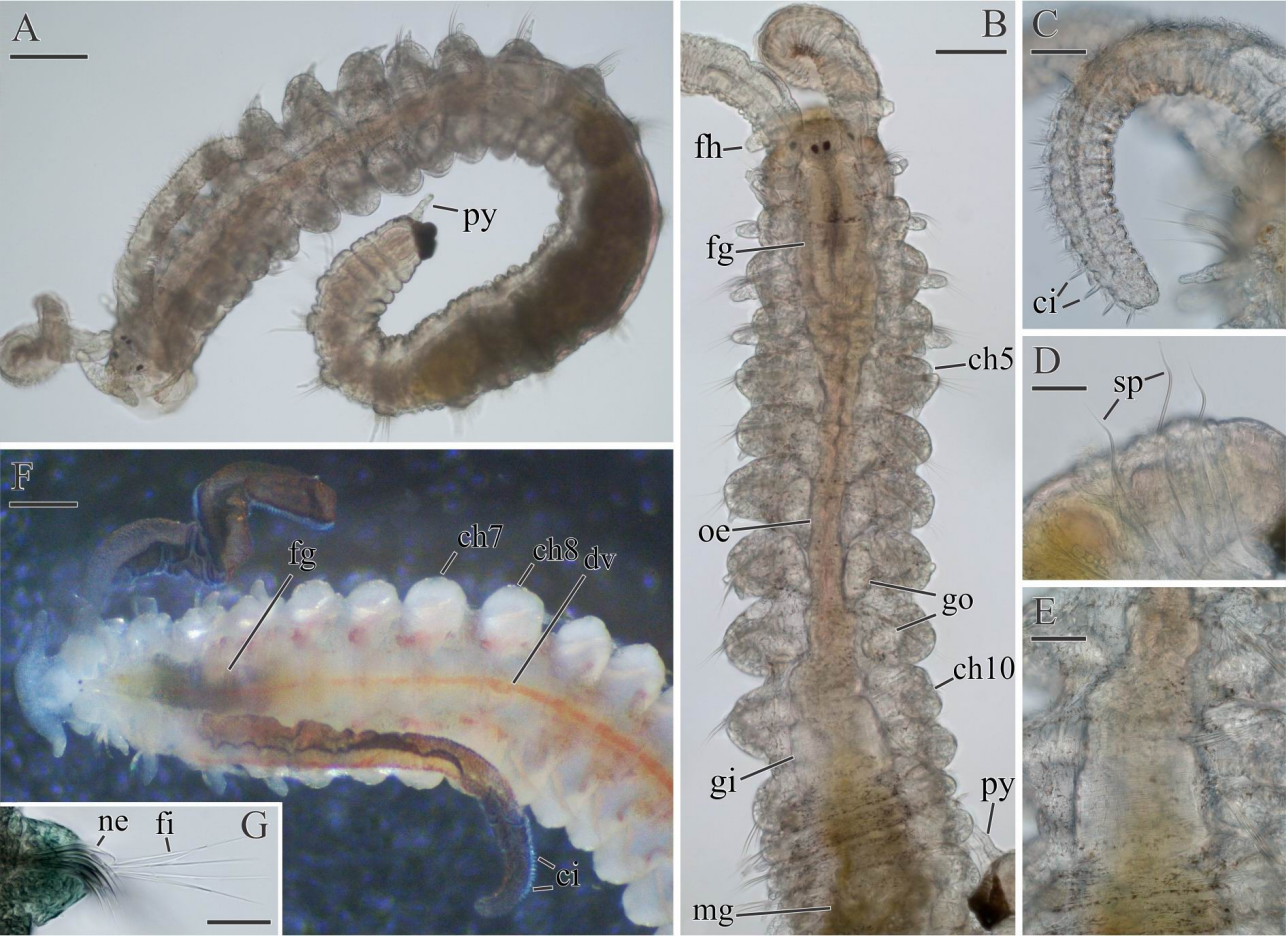
Juvenile and adult morphology of *Spiophanes hakaiensis* n. sp. (A–E)–live 24-chaetiger juvenile. (A) Complete individual. (B) Anterior end and pygidium, dorsal view. (C) Right palp, frontal view. (D) Posterior chaetigers, left lateral view. (E) Gizzard-like structure, dorsal view. (F) Anterior end of a live adult individual, dorsal view. (G) Left neuropodium of chaetiger 6 of a formalin-fixed 40-chaetigers juvenile, ventral view, showing short neurochaetae and long thin straight fibers protruding from hole-like opening of a glandular organ. Abbreviations: *ch5–ch10* –chaetiger 5–10; *ci*–transverse rows of short motile compound cilia on inner lateral side of palp; *dv*–main dorsal blood vessel; *fg*–foregut; *fh*–fronto-lateral horn; *fi*–fibers produced by glandular organs; *gi*–gizzard-like structure; *go*–glandular organs; *mg*–midgut; *ne*–capillary neurochaetae; *oe*–oesophagus; *pg*–posterior gut; *py*–pygidial cirri; *sp*–notopodial spines with recurved distal end. Scale bars: A, B– 100 μm; C–E, G– 50 μm; F– 300 μm. A–E–British Columbia, Canada, MIMB 36708, F–British Columbia, Canada, CMNA 2019–0105 (holotype). G–Oregon, USA, MIMB 36711.

*Spiophanes norrisi*: Meißner & Blank, 2009 [111] (*Part*.): 11–15.

*Spiophanes bombyx*: Berkeley [53]: 416; [179]: 560. Berkeley & Berkeley [57]: 22–24, Figs 40–43. Hobson & Banse [62]: 45, Fig 6l. Carey [180]: 438. Macdonald *et al*. [181]: 40. Carr [46]: Supplement. Not Claparède [9, 10].

*Type material*. CANADA, **British Columbia**, 2017 Hakai-MarineGEO BioBlitz, Queen Charlotte Sound, Calvert Is.: Choked Pass, North Pigu Is., st. IHAK-46B, 51.6763°N, 128.1222°W, 6 m, sea grass, coll. D. VanMaanen, 4 Aug 2017, CMNA 2019–0105 (holotype); Choked Pass, st. IHAK-42B, 51.6806°N, 128.1163°W, 5 m, outer sandspit sediment, coll. G. Paulay, 3 Aug 2017, MIMB 36708 (paratype); Pruth Bay, st. SHAK-10, 51.6441°N, 128.1195°W, low intertidal, muddy sand, eel grass, coll. S. Dudas, 24 Jul 2017, MIMB 36707 (paratype). USA, **California**, offshore San Francisco: st. SWOO-63, 37.6588°N, 122.5615°W, 27.5 m, coll. CCSF/SFPUC Lab, 13 Sep 2010, MIMB 39021 (13 paratypes); st. SWOO-73, 37.7125°N, 122.5648°W, 18.5 m, muddy sand, 13 Sep 2010, MIMB 39022 (2 paratypes).

**Adult morphology** (based on the type material–specimens identified by means of molecular analysis in the present study). Up to 25 mm long, 1.3 mm wide for 110 chaetigers. Pigmentation absent on body. Few examined live complete individuals with fine black pigment scattered on palps (Fig 16F). In large formalin-fixed specimens, lateral sides of chaetigers 9–14 (until chaetigers 16–17 in largest specimens) light reddish due to fixed internal content of large, probably glandular, epithelial cells.

Prostomium triangular, wide anteriorly, with a pair of long, distally pointed fronto-lateral horns (Fig 16A, 16B and 16F), posteriorly narrowed, pressed into chaetiger 1 but not extending over as a caruncle. Peristomium with narrow lateral lips closely applied to lateral sides of prostomium, and a small ventral lip. Two pairs of small dark red eyes arranged trapezoidally, comprising one pair of median eyes and one pair of lateral eyes situated anteriorly and set wider apart; occasionally eyes absent. Occipital antenna absent. Palps as long as 7–15 chaetigers, with deep frontal longitudinal groove lined with fine cilia, fronto-lateral motile compound cilia bordering frontal groove, short transverse rows of short motile compound cilia regularly arranged on inner lateral side and beating towards frontal groove (Fig 16C and 16F), short compound non-motile cilia arising directly from palp surface and scattered on lateral and abfrontal palp surfaces.

Nuchal organs metameric, at least on 25 anterior chaetigers, fewer in small individuals. First pair of metamers long oblique ciliary bands extending from posterior part of prostomium to end of chaetiger 2, shorter in small individuals; posterior ends of metamers set wider apart. Succeeding metamers shorter, each extending from nototroch over posterior half of chaetiger, oriented parallel to body axis until chaetigers 10–11, oblique on succeeding chaetigers.

Notochaetae capillaries, long on four anterior chaetigers and on posterior chaetigers, shorter on middle chaetigers. Neuropodia of chaetiger 1 each with 1–2 heavy recurved, crook-like spines in addition to capillaries. Notopodia of 5–10 posterior chaetigers each with 1–3 (usually one) long spines with recurved distal end in addition to capillaries (Fig 16D). Noto- and neuropodial postchaetal lamellae on four anterior chaetigers subtriangular, with pointed tips; notopodial lamellae on succeeding middle chaetigers short, subulate, on posterior chaetigers long, cirriform. Neuropodial postchaetal lamellae on chaetigers 5–14 large but low, rounded and fleshy, with openings of internal glandular organs.

Sabre chaetae in neuropodia from chaetiger 10, usually one, occasionally two per neuropodium, with fine granulation on distal part; chaetae on chaetiger 10 comparatively small, with narrow limbation, slightly reducing in size until chaetiger 14, but from chaetiger 15 onwards thicker, alimbate.

Hooks in neuropodia from chaetiger 15, up to 10 in a series, accompanied by inferior sabre chaetae throughout body and 1–2 hair-like alimbate capillaries in 5–10 posterior chaetigers. Hooks usually quadridentate, with three small upper teeth above main fang and small subterminal hood below main fang.

Nototrochs from chaetiger 1 to end of body, each composed of two parallel rows of large transversally elongated cells, each bearing numerous long cilia; nototroch ciliation stronger on ridge-bearing chaetigers. Intersegmental transverse ciliation from chaetiger 3 onwards.

Dorsal transverse ridges from chaetigers 14–15 to chaetigers 30–55, fewer in small individuals. Intersegmental lateral pouches absent.

Pygidium with small fleshy ventral pad and one pair of thin long lateral cirri.

Glandular organs in chaetigers 5–14, largest in chaetigers 7 and 8, smallest in chaetiger 9 and gradually increasing in size from chaetiger 10 to chaetigers 12–13, slightly smaller again in chaetiger 14; large organs occupying most of chaetiger space. Internal fibres long and coiled in glandular organs in chaetigers 5–8, straight, shorter and thinner in chaetigers 9–14; in fixed specimens, long straight fibers usually protruding from openings of glandular organs on chaetiger 6 (Fig 16G). Each organ on chaetigers 5, 7 and 8 opening to exterior via semicircular to suboval slit around large fiber spreader, on chaetiger 6 via small round hole, and on chaetigers 9–14 via large vertical slit. Frontal edge of each fiber spreader on chaetigers 5, 7 and 8 blunt or with variously developed middle depression vaguely separating two rounded lobes.

Foregut wide, eversible, within 3–4 anterior chaetigers, with greenish-black pigment in thick wall; oesophagus in succeeding chaetigers narrow, with thin unpigmented wall (Fig 16F). Gizzard-like structure in end of oesophagus beginning from chaetigers 10–20 and extending through 2–3 chaetigers (Fig 16B–16E). Posterior gut with transparent wall.

Main dorsal blood vessel with heart body extending from chaetigers 12–15 to chaetigers 19–20. Blood red, without elements (Fig 16F).

Nephridia from chaetiger 15 onwards, opening to exterior on antero-lateral edges of chaetigers in both sexes.

*Juvenile morphology*. 23-chaetiger juvenile about 2 mm long, 0.3 mm wide on chaetiger 5, with fronto-lateral horns on prostomium well developed (Fig 16A). First pair of nuchal metamers extending to middle of chaetiger 2. Succeeding metamers on chaetigers 6–11. Transverse rows of short motile compound cilia regularly arranged on distal half of each palp (Fig 16C). Double-row nototrochs from chaetiger 3 onwards. Sabre chaetae in neuropodia from chaetiger 11, very small on the first chaetiger, better discernible from chaetiger 12. Hooks in neuropodial from chaetiger 14. Long spines with recurved distal end in notopodia of chaetigers 19–23 (Fig 16D). Pygidium with ventral semispherical yellow pad and one pair of thin transparent dorsal cirri (Fig 16A and 16B). Gizzard-like structure in chaetigers 10–11. Nephridia from chaetiger 15 onwards.

30–40-chaetiger juveniles about 0.4 mm wide with sabre chaetae in neuropodia from chaetiger 10, 1–2 hooded hooks among 4–6 capillary chaetae and single sabre chaeta in each neuropodium of chaetiger 14, and only hooks and sabre chaetae in neuropodia from chaetiger 15 onwards. Individuals more than 0.4 mm wide with morphological characters as described above in Adult morphology, with sabre chaetae in neuropodia from chaetiger 10 and hooded hooks from chaetiger 15.

*Methyl green staining*. Intensely stained lateral sides of chaetiger 6 and small fleshy pygidium (pygidial cirri not stained), weakly stained lateral sides of chaetigers 9–14; no staining on chaetigers 1–5 and 7, 8 (Fig 10D).

*Reproduction*. *Spiophanes hakaiensis* n. sp. is gonochoristic. Mature females and males collected in British Columbia, Canada, in May 1966 (RBCM 55–47), have gametes from chaetiger 21 through most of the body. The oocytes have honey-combed envelopes with cortical alveoli regularly arranged in a peripheral circle. Spermatids are interconnected in tetrads; the spermatozoa were ect-aquasperm with a short acrosome and short, subspherical nucleus about 3 μm in diameter.

*Remarks*. Horned *Spiophanes* from the North East Pacific appear morphologically very similar or almost identical to each other and to *S*. *uschakowi* from the North West Pacific. However, molecular analysis performed in the present study showed significant difference between the North East and North West populations and also suggested the presence of two sibling vicariant species distributed along the Pacific coast of North America and overlapping in California (Figs 1B, 2B and 3). Sequences of some Californian specimens (including *COI* sequences provided by Meißner & Blank [111]) were grouped together with sequences of specimens from Baja California Norte, Mexico (see Table 1, Fig 1B). Because the latter locality is close to the type locality of *S*. *norrisi*, those specimens are referred to this species. Other Californian specimens were genetically similar to horned *Spiophanes* from Alaska, USA, and British Columbia, Canada. Those specimens are herein referred to a new species: *Spiophanes hakaiensis* n. sp. Comments on the taxonomy and the distribution of these species are provided above in the Remarks for *S*. *norrisi*, and below in the Remarks for *S*. cf. *norrisi*.

To avoid confusion in the identification of specimens based on the morphology only, in areas of overlap of *S*. *hakaiensis* n. sp. and *S*. *norrisi*, we suggest that specimens from California between Point Reyes (38°N) and Point Conception (34.45°N) are referred to as *S*. *norrisi* aggregate; specimens occurring from Point Reyes north to Alaska are herein referred to as *S*. *hakaiensis* n. sp.; whereas those occurring from Point Conception south to Baja California Sur, Mexico, are referred to as *S*. *norrisi*.

*Distribution*. From Alaska south to San Francisco Bay, California, USA (Fig 15A).

***Spiophanes* cf. *norrisi* Meißner & Blank, 2009.** Fig 10C

*Spiophanes bombyx*: Hartman [109]: 22, pl V, Figs 14–16. Carrasco [67]: 197–199, Figs 37–41; [119]: 48–53, Figs 19, 20, 22 J–L (larval morphology). Blake [48]: 230. Rozbaczylo & Salgado [71]: 23, Fig 2e. Carrasco [182]: 453, 455. Mendez *et al*. [183]: 431. Montiel *et al*. [73]: 310. Not Claparède [9, 10].

*Spiophanes chilensis*: Hartmann-Schröder [184] (*Part*.): 215–218.

*Spiophanes norrisi*: Meißner & Blank [111] (*Part*.): 11–15, Fig 6C.

*Remarks*. Hartmann-Schröder [184] described Chilean *Spiophanes* (distributed from Punta Tortuga, Coquimbo, south to Gulf of Corcovado) with prostomium expanded anteriorly as *S*. *chilensis* Hartmann-Schröder, 1965. Meißner [8] and Meißner & Blank [111] re-examined the type material of *S*. *chilensis* and distinguished specimens belonging to three species: *Spiophanes duplex* (Chamberlin, 1919), *Spiophanes fimbriata* Moore, 1923, and *S*. *norrisi*. Meißner [8] referred the holotype of *S*. *chilensis* to *S*. *duplex* and considered the former species as a junior synonym of the latter (see below). Meißner & Blank ([111]: 14–15) referred 22 paratypes of *S*. *chilensis* from Punta Tortuga (29°57.350' S, ZMH P-14946) and additional material from Bahia Quillaipe (41°32.469' S, ZMH P-21118, 2 spec.) to *S*. *norrisi* and concluded that the latter species “occurs from shallow waters up to subtidal depths waters along the North and South American coast.”

Meißner & Blank [111] did not mention the records of *S*. *bombyx* from Chile by Carrasco [67, 119] and from Argentina and the Falkland Islands by Blake [48], although these two authors noticed that their specimens were identical with *S*. *bombyx* from Europe. We also had no chance to examine Carrasco’s or Blake’s material but got new material from Chile and Argentina (see S1 Table). We could not find any difference between this material and corresponding specimens from the Pacific USA and Canada, either in the morphology or in the MG staining (Fig 10C–10E). Nevertheless, pending results of a comparative molecular analysis of North and South American specimens (see above Remarks for *S*. *norrisi*), we suggest that Chilean and Argentinean specimens are referred to as *S*. cf. *norrisi*. It is quite possible that South American horned *Spiophanes*, although morphologically appearing similar to North American *S*. *norrisi*, differ from them genetically and represent a different species. We see support to this idea in the limited distribution of worms in the southern part of South America only.

**Fig 17:**
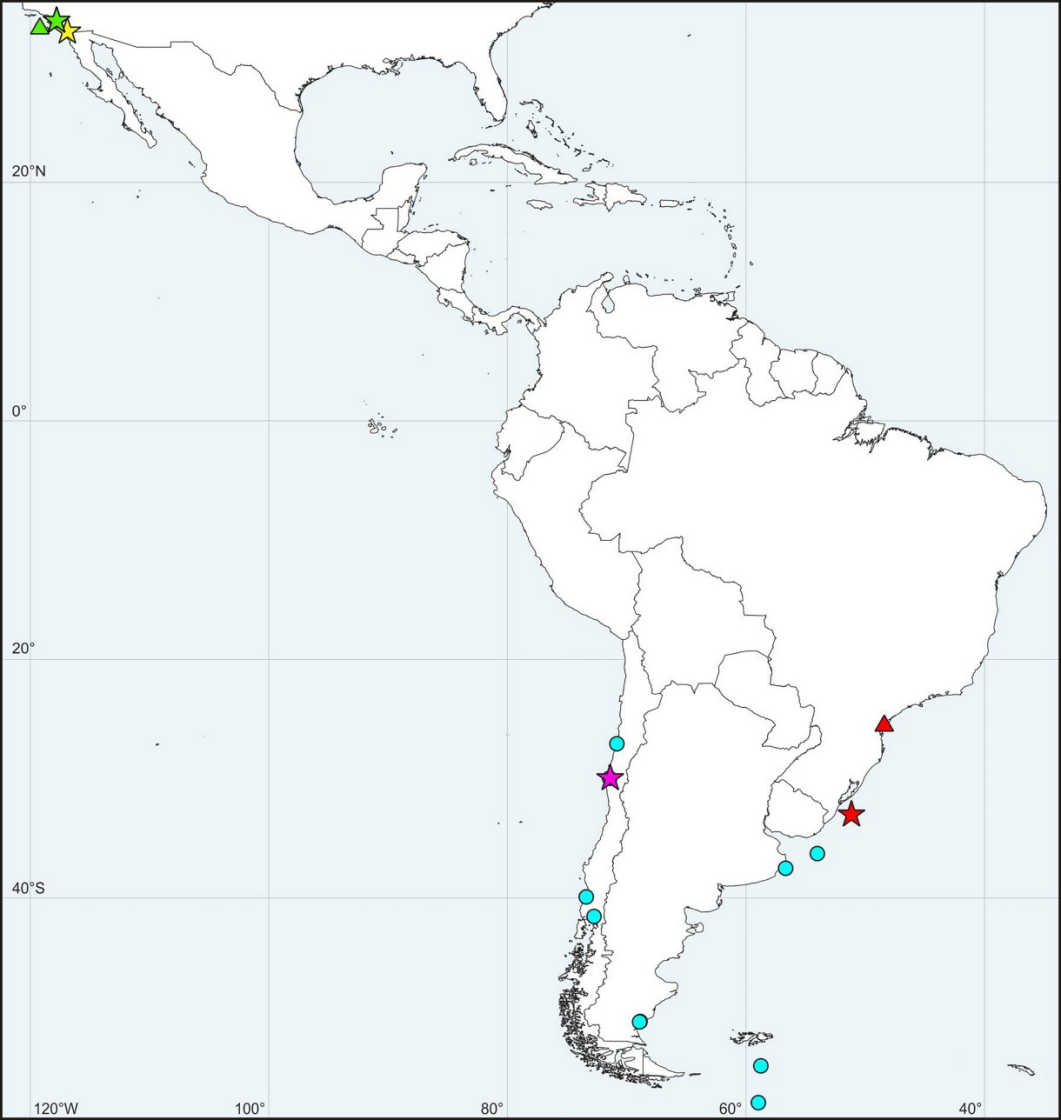
Map showing type localities and records of *Spiophanes* spp. in North and South Americas.

*Distribution*. South America: Chile, Argentina, Falkland Islands (Fig 17).

***Spiophanes duplex*** (Chamberlin, 1919) (green and yellow symbols): green star–type locality: Laguna Beach, California, USA; green triangle–specimens sequenced in the present study; yellow star–type locality of ***Spiophanes missionensis*** Hartman, 1941 (junior synonym of *S*. *duplex*): Mission Bay, California, USA. ***Spiophanes soederstroemi*** Hartman, 1953 (red and pink symbols): red star–type locality: off Rio Grande do Sul, Brazil; red triangle–specimens sequenced in the present study; pink star–type locality of ***Spiophanes chilensis*** Hartmann-Schröder, 1965 (junior synonym of *S*. *soederstroemi*): Punta Tortuga near Coquimbo, Chile. ***Spiophanes* cf. *norrisi***: turquoise circles–reports as *S*. *bombyx* by Blake [48], Mendez *et al*. [183], Meißner & Blank [111], and identified in the present study based on the morphology only.

*Spiophanes duplex* (Chamberlin, 1919)

*Morants duplex* Chamberlin, 1919 [185]: 17. *Fide* Blake [64]: 149.

*Spiophanes missionensis* Hartman, 1941 [186]: 296–298, pl 46, Figs 17–21; [56]: 46; [187]: 46. Reish & Winter [188]: 113. Barnard & Reish [189]: 88. Reish & Barnard [190]: 5. Reish [191]: 84; [192]: 77. Kauwling & Reish [193]: 58. Hernández-Alcántara *et al*. [63]: 570. Maurer *et al*. [194]: 185–203. Méndez [176]: 142. Salazar-Vallejo & Londoño-Mesa [195]: 61. *Fide* Blake [64]: 149.

*Spiophanes duplex*: Blake [64]: 149–150, Fig 4.20; [178]: 592–593, Fig 13.11 (larval morphology). Hernández-Alcántara & Solís-Weiss [175]: 29; [196]: 285. Salazar-Vallejo & Londoño-Mesa [195]: 61. Meißner [8] (*Part*.): 31–36. Blake & Ruff [197]: 377. Díaz-Castañeda & Valenzuela-Solano [177]: 513. Schiff *et al*. [198]: 41–42.

*Type material*. USA, **California**, Orange Co., Laguna Beach, Balboa, 33.5419°N, 117.7892°W, intertidal, MCZ ANNb-2165 (holotype).

*Remarks*. *Spiophanes duplex* was originally described from Laguna Beach, Balboa, California, USA, by Chamberlin ([185], as *Morants duplex*). The original description was brief, with some features misinterpreted, without any illustration. Misinterpreted features greatly distinguished these worms from other spionids, and based on them, Chamberlin [185] established a new genus, *Morants* Chamberlin, 1919. In the *Catalogue of the Polychaetous Annelids of the World*, Hartman [1999] noted *Morants* as a monotypic genus. The genus and the species have been enigma for many years until Blake [64] discovered the holotype of *M*. *duplex* in the collections of the Museum of Comparative Zoology at Harvard (MCZ 2165) and redescribed it. He noted that the holotype had all the characters of *Spiophanes* and referred to *Morants* as a junior synonym of *Spiophanes*. Blake [64] also referred to *Spiophanes missionensis* Hartman, 1941, originally described from Mission Bay, San Diego County, California, USA, as a junior synonym of *S*. *duplex*.

Meißner [8] reexamined the type specimens of *S*. *missionensis* Hartman, 1941, *S*. *soederstroemi* Hartman, 1953, and holotype of *S*. *chilensis* Hartmann-Schröder, 1965 and, based on their morphological similarity, considered these species as junior synonyms of *S*. *duplex* (Chamberlin, 1919). Consequently, she suggested that *S*. *duplex* is widely distributed along both the Pacific and Atlantic coasts of both North and South America.

The results of the present study unambiguously show that specimens from Brazil, being morphologically identical to adults of *S*. *duplex* from California, differ from them essentially in genetic characteristics (Fig 2B). Therefore, we considered the two populations as not conspecific and refer the Brazilian population to *S*. *soederstroemi* (see below). Pending results of molecular studies in other American regions, we consider *S*. *duplex* as distributed in California only.

*Distribution*. North East Pacific: California (Fig 17).

***Spiophanes soederstroemi* Hartman, 1953.** *Spiophanes kroyeri*: Söderström [21] (*Part*.): 243. Not Grube [200]. *Fide* Hartman [201]: 41.

*Spiophanes soederstroemi* Hartman, 1953 [201]: 41, Fig 14. Orensanz & Gianuca [202]: 17–18. Blake [48]: 230–232, Fig 13. Rozbaczylo & Castilla [203]: 175. Rozbaczylo & Salgado [71]: 24–25, Fig 2f, g. Carrasco [182]: 453, 455. Cañete *et al*. [204]: 247. Maciolek [205] (*Part*.): 543–544. Lancellotti & Stotz [206]: 309. Palma *et al*. [207]: 240. Montiel *et al*. [73]: 310.

*Spiophanes chilensis* Hartmann-Schröder, 1965 [184] (*Part*.): 215–218, Figs 208–211. *Fide* Blake [48]: 230.

*Spiophanes missionensis*: Bolivar & Lana [49]: 132–134, Figs 48–53; [50]: 255. Morgado & Amaral [208]: 549. Paiva [209]: 42; [210]: 74. Muniz & Pires [211]: 520; [51]: 152. Arasaki *et al*. [212]: 258. Santi & Tavares [213]: 293. Not Hartman [186].

*Spiophanes duplex*: Meißner [8] (*Part*.): 31–36, Figs 16–19. Pardo *et al*. [52]: 220, textfigs A–F. Scarabino [214]: 121. Pagliosa *et al*. [215]: 46. Radashevsky [174]: Fig 12A. Shimabukuro *et al*. [216]: 9. Not Chamberlin [185].

*Spiophanes* sp.: Radashevsky [174]: Figs 4A, 10C, 10D.

*Type material*. Off Brazil, **Rio Grande do Sul**, Swedish Antarctic Expedition 1901–1903, st. 1, 33.00°S, 51.1667°W, 80 m, bottom of dark gray mud, 12 Dec 1901, SMNH 5369 (2 syntypes).

*Remarks*. *Spiophanes soederstroemi* Hartman, 1953 was established by Hartman [201] based on two specimens from South America collected by the Swedish Antarctic Expedition 1901–1903 onboard the ship *Antarctic*. The specimens were first reported by Söderström [21] as a new record for *S*. *kroyeri*. Remarkably, Söderström ([21]: 243) and all the following authors noted that the specimens were collected on December 12, 1901 at station 1, off Uruguay. However, the coordinates provided for this station by Söderström [21] and also in a copy of the actual station list made during the expedition: 33°00'S, 51°10'W (SMNH, Lena Gustavsson *in litt*., 11 Sep 2019), and a map of the Expedition route with daily positions of the ship [217] show a sampling site of December 12, 1901 situated off Rio Grande do Sul, Brazil (Fig 17), north of the border between Brazil and Uruguay. Noteworthy, although this border was already well established in the mid 19^th^ century and did not change after that, it was not shown on the Expedition map. Possible uncertainty in its exact position might create confusion about reference on the type locality of *S*. *soederstroemi*.

Blake [48] redescribed two syntypes of *S*. *soederstroemi*, reviewed a complicated history of this species and clarified some of its morphological features and taxonomic issues. He also reexamined the type specimens of *Spiophanes chilensis* Hartmann-Schröder, 1965, which was originally described from Punta Tortuga, Chile, by Hartmann-Schröder [184], and referred its holotype (ZMH P-14946) to *S*. *soederstroemi*. Consequently, Blake [48] considered *S*. *chilensis* as a junior synonym of *S*. *soederstroemi*. He also noticed subtle differences between *S*. *soederstroemi* and *S*. *missionensis* and considered them as two closely related but separate species (the holotype of *Morants duplex* was not yet discovered at that time).

Meißner [8] reexamined type specimens of *S*. *missionensis* Hartman, 1941, *S*. *soederstroemi* Hartman, 1953, and holotype of *S*. *chilensis* Hartmann-Schröder, 1965 and, based on their morphological similarity, considered these species as junior synonyms of *S*. *duplex* (Chamberlin, 1919).

During the present study, we examined live *Spiophanes* in California, USA, and Paraná, Brazil, which fit all the characteristics of *S*. *duplex*. We could not find any reliable morphological difference between them, but the differences between their molecular characteristics suggest the presence of two distinct species (Fig 2B). Consequently, we treat *S*. *duplex* and *S*. *soederstroemi* as two valid species, and consider *S*. *missionensis* as a junior synonym of *S*. *duplex*, and *S*. *chilensis* as a junior synonym of *S*. *soederstroemi*. The type localities of these species and sampling sites for the specimens used in the present molecular analysis are shown on Fig 17.

*Spiophanes soederstroemi* is likely distributed in South America only. Records of *S*. *soederstroemi* from South Africa [100, 101], South Georgia [109], Antarctica [218, 219], China [90], Florida and North Carolina, USA [205], Philippines [220], and the Arabian Gulf [221] possibly represent misidentifications of different species.

*Distribution*. South America: Brazil, Uruguay, Argentina, Chile (Fig 17).

***Spiophanes kroyeri* Grube, 1860.** *Spiophanes kroyeri* Grube, 1860 [200]: 88–89, pl V, Fig 1. Sikorski [33] (*Part*.): 329–331, textfig Meißner [8] (*Part*.): 7–14, Fig 1A.

*Type material*. Greenland Sea, off northern Iceland, coll. Grube, E., ZMB Q4746 (lectotype), 11168 (paralectotype).

*Remarks*. Grube ([200]: 89) noted in the original description of *S*. *kroyeri* that the new species was “Aus dem Meere von Grönland.” No more detail about the type locality has ever been published, but the original registry catalogue and labels for the types of *S*. *kroyeri* maintained in the polychaete collection of the Zoologisches Museum Berlin (ZMB), Germany, say that the material was from Iceland, *i*.*e*. from the southern part of the Greenland Sea ([222]; Birger Neuhaus *in litt*., 15 Jul 2019). A tentative position of the type locality is shown on the Fig 4B.

Meißner ([8]: 13) considered *S*. *kroyeri* as “the most problematic species within the genus.” She reviewed its worldwide reports and suggested that most of them were based on misidentifications. To clarify the identity of *S*. *kroyeri*, Meißner [8] designated a lectotype (ZMB Q4746) and provided a series of diagnostic characteristics of the adults. They included a bell-shaped prostomium, well developed occipital antenna, nuchal organs as a pair of longitudinal ciliary bands extending to chaetigers 14–16, suboval chaetal spreaders well developed on chaetigers 5–7, sabre chaetae in neuropodia from chaetiger 4, hooks with subterminal hood in neuropodia from chaetiger 15, lateral pouches first appearing between neuropodia of chaetigers 15 and 16, and pigmentation comprising light brown pigment in postchaetal lamellae of chaetigers 1–7 and brown pigment in neuropodia of chaetigers 7–14, most conspicuous in chaetigers 8–11.

Meißner ([8]: 14) commented on the problems with the original description and type material of *S*. *kroyeri reyssi* described from Mediterranean France by Laubier [223]. She raised it to species level, *S*. *reyssi* Laubier, 1964, and emphasized that “so far only specimens from the North Atlantic can be assigned to *S*. *kroyeri*”.

The analysis of *COI* sequences of *Spiophanes* from the North Sea obtained by Meißner & Blank [111], from the Bay of Biscay, Spain, obtained by Aylagas *et al*. [121], and from the Barents Sea, Norway, obtained in the present study (see Table 1), revealed two distinct groups among specimens identified by morphology as *S*. *kroyeri* (Fig 1B). Worms of one group occurred in the Barents Sea, Norway, and in the northern part of the North Sea (vouchers HH64, 94 by Meißner & Blank [111]), whereas worms of another group occurred in the northern (vouchers HH18, 19 by Meißner & Blank [111]) and central parts of the North Sea (voucher HH63 by Meißner & Blank [111]), and in the Bay of Biscay, Spain. According to their distributions, we call these groups northern and southern, respectively. In relation to the type localities of *S*. *kroyeri* (Greenland Sea, off northern Iceland) and *S*. *cirrata* (Oslofjord, southern Norway, see below), we tentatively refer to the northern-group specimens as *S*. cf. *kroyeri*, and to the southern-group specimens as *S*. cf. *cirrata* (Fig 4B). Final conclusion about the specific identity of these worms can be inferred after molecular examination of worms from the type localities of the corresponding species in Iceland, Norway, and Mediterranean France. To avoid confusion in the identification of *S*. *kroyeri*-like specimens from the northern part of the North Sea (area of overlap of the two species) based on morphological characters only, we suggest that they are referred to as *S*. *kroyeri* aggregate (*S*. *kroyeri* agg.), rather than to a particular species.

It is noteworthy that deep-water *Spiophanes* worms from off the Crozet Islands, Southern Ocean, referred to as *S*. *kroyeri* by Mincks *et al*. [122], are genetically different from European specimens (Fig 2B) and are therefore referred to herein as *S*. aff. *kroyeri*.

*Distribution*. Arctic; North Atlantic (Fig 4B).

***Spiophanes cirrata* M. Sars in G.O. Sars, 1872.** *Spiophanes cirrata* M. Sars in G.O. Sars, 1872 [224]: 410–411. M. Sars [225]: 268–273, pl XVIII, Figs 1–16.

?*Spiophanes kroyeri*: Hannerz [163]: 36–40, Figs 10, 11 (larval morphology). Eliason [19]: 49–50; [20]: 263. Hartmann-Schröder [164]: 326–327, Fig 111; [165]: 342–343, Fig 157. Böggemann [32]: 121, Fig 98. Jelsing [226]: 244, 247, Fig 1I (nuchal organs). Meißner [8] (*Part*.): 7–14, Figs 1B–H, 2, 3. Zettler *et al*. [227]: checklist. Not Grube [200].

Not *Spiophanes cirrata*: Berkeley [53]: 416; [179]: 560. Berkeley & Berkeley [228]: 475, textfig; [57]: 24–25, Figs 44–46; [229]: 791. Carey [180]: 439.

*Type material*. NORWAY, North Sea, Skagerrak, Oslofjord, Drøbak, types missing, Oslo, Norway.

*Remarks*. Michael Sars (in G.O. Sars [224]) briefly described *Spiophanes cirrata* based on material from Drøbak, Oslofjord, southern Norway (Fig 4B), and Skrova, small island group in the Lofoten Archipelago, northern Norway. Later, Sars [225] illustrated and provided a more complete re-description of this species. Tauber [230] and Söderström [21] considered *S*. *cirrata* to be a junior synonym of *S*. *kroyeri*. Hartman [199] listed *S*. *cirrata* as a valid species. Hartmann-Schröder [164, 165] and Kirkegaard [231] neither noted nor commented on this species. As the first sampling site mentioned in the original description of the species by Michael Sars, Drøbak in Oslofjord is herein for the first time recognized as the type locality of *Spiophanes cirrata*.

Based on the results of the molecular analysis performed in the present study (see above Remarks for *Spiophanes kroyeri*), and according to the type localities of *S*. *kroyeri* and *S*. *cirrata*, we tentatively refer *S*. *kroyeri*-like worms from the Barents Sea, Norway, and the northern part of the North Sea to as *S*. cf. *kroyeri*, and those from the northern and central parts of the North Sea, and from the Bay of Biscay, Spain, to as *S*. cf. *cirrata* (Fig 4B). Final conclusions about the specific identity of these worms can only be inferred after molecular examination of worms from the corresponding type localities of these species.

*Distribution*. North East Atlantic (Fig 4B).

***Spiophanes berkeleyorum* Pettibone, 1962.** *Spiophanes berkeleyorum* Pettibone, 1962 [232]: 78–83, Figs 1–4. Light [60]: 78–79, Fig 5a, b; [61]: 63–66, textfigs 63–65. Hobson & Banse [62]: 45, Fig 6k. Blake [64]: 143–145, Fig 4.17. Meißner [8]: 28–31, Figs 13–15. Blake & Ruff [197]: 377. Carr [46]: Supplement.

*Spiophanes cirrata*: Berkeley [53]: 416; [179]: 560. Berkeley & Berkeley [228]: 475, textfig; [57]: 24–25, Figs 44–46; [229]: 791. Carey [180]: 439. Not M. Sars in G.O. Sars [224]. *Fide* Pettibone [232].

*Type material*. CANADA, **British Columbia**, east coast of Vancouver Island, Departure Bay beach, coll. E. & C. Berkeley, 25 Apr 1936, USNM 30399 (holotype), 30400 (6 paratypes).

*Remarks*. *Spiophanes berkeleyorum* was originally described from Vancouver Island, British Columbia, Canada, by Pettibone [232] and later reported from distant regions. Meißner & Hutchings [110] removed *Spiophanes japonicum* Imajima, 1991 from synonymy with *S*. *berkeleyorum* and Meißner [8] suggested that the latter species is distributed along the Pacific coast of North America only. Later reports of the species from Brazil [233, 215], Russia [84], and China [95] probably represent misidentifications and should be verified in additional studies. Molecular data of *S*. *berkeleyorum* are limited to sequences provided by Meißner & Blank [111] and those in the present study.

*Distribution*. North East Pacific: from Alaska south to California.

***Trochochaeta multisetosa* (Örsted, 1843).** *Disoma multisetosum* Örsted, 1843 [234]: 41–42; [235]: 107–108, pl II, Figs 1–12. Thulin [236]: 9, Figs 7–17. Ditlevsen [237]: 32. Friedrich [23]: 135, Fig 88. Wesenberg-Lund [238]: 31, pl 7, Figs 31–32. Hannerz [163]: 141, Figs 51–52. *Fide* Pettibone [239]: 310.

*Trochochaeta multisetosa*: Pettibone [239]: 310–315, Figs 82, 83 a–g.

*Trochochaeta multisetosum*: Weitbrecht [240]: 401–412, Figs 1–10.

*Disoma franciscanum* Hartman, 1947 [241]: 160–169, Figs 1–3. *Fide* Pettibone [239]: 310.

*Trochochaeta franciscanum*: Blake & Arnofsky [242]: 68, Fig 6. Blake [178]: 598–600, Fig 13.13. Blake & Ruff [197]: 377.

*Trochochaeta franciscana*: Radashevsky *et al*. [243]: 574–575.

*Type material*. SWEDEN, Near Hveen (Ven) Island in the Øresund Strait. Types missing.

*Remarks*. Although *Trochochaeta* was not an object of the present study, use of these worms in the analysis (as an outgroup) brought some insight on their taxonomy. *Trochochaeta multisetosa* was originally described from the Øresund Strait, Sweden, by Örsted ([234], as *Disoma multisetosum*) and later occasionally reported from the North East Atlantic. Hartman [241] described *Trochochaeta franciscana* (as *Disoma franciscanum*) from San Francisco Bay, California, USA, but Pettibone [239] placed it into synonymy with *T*. *multisetosa*. In disagreement with Pettibone [239], Blake & Arnofsky ([242]: 68) stated that “the California specimens clearly represent a separate species due to differences in larval ciliary patterns.” In a revision of *Trochochaeta*, Radashevsky *et al*. [243] treated *T*. *multisetosa* and *T*. *franciscana* as two valid species. Molecular analysis performed in the present study, however, showed identity (*p-*distance = 0.00%) of the fragments of nuclear *18S* and *28S* rDNA sequenced in worms from Norway and Sweden and from Drake’s Bay, California, adjacent to San Francisco Bay. Consequently, in agreement with Pettibone [239], we tentatively suggest that *T*. *franciscana* is a junior synonym of *T*. *multisetosa*. As such, it may represent an introduced North Atlantic species in San Francisco Bay (where it was first collected in 1912, Hartman [241]) and nearby embayments, transported by vectors such as commercial oyster culture or shipping. This suggestion should be verified, however, by analyzing mitochondrial genes, such as *COI* and *28S* rDNA, of individuals from the San Francisco Bay area, and by further analysis of additional populations, reported under the names of *T*. *franciscana* or *T*. *multisetosa* from a variety of habitats, from British Columbia to southern California, to further confirm if only one species of *Trochochaeta* is present.If confirmed, this may be a second case of the successful invasion of *Trochochaeta* into a remote region. Radashevsky *et al*. [243] recently suggested that *Trochochaeta japonica* Imajima, 1989 might have been introduced from the Asian Pacific to the estuary of Santos, São Paulo, Brazil, as larvae in ballast water of ocean-going vessels.

## Discussion

The molecular analyses performed in the present study provided rather unexpected results. In various cases, morphologically similar or even identical specimens from close locations or even from the same area turned out to be genetically distant and are herein referred to as members of sibling species.

### Five-genes analysis (*COI*, *16S*, *18S*, *28S*, and *Histone 3*)

The five-genes analysis unambiguously suggested that horned *Spiophanes* with metameric nuchal organs and *Spiophanes* with bell-shaped prostomia and nuchal organs as entire long parallel ciliary bands belong to two major evolutionary lineages within the genus. More comprehensive analysis including more species is required to confirm this hypothesis. Including species with other types of nuchal organs, such as entire U-shaped ciliary bands (see Radashevsky [174] for review), in a future analysis, will help to better understand the evolutionary history of *Spiophanes* and the transformation of characters within this group of spionids. Remarkably, adults of *Trochochaeta*, the so far suggested sister group to *Spiophanes*, have U-shaped nuchal organs, similar to those of some *Spiophanes* and many other spionids. It will not be surprising, therefore, if *Spiophanes* with U-shaped nuchal organs are shown to have a basal position on the phylogenetic tree of the genus. This would suggest that the metameric and nuchal organs as long parallel ciliary bands might have evolved from nuchal organs as U-shaped ciliary bands within *Spiophanes*. This would also suggest that the metameric and nuchal organs as long parallel ciliary bands shared by other spionids, might be homoplasious characters evolved independently more than once within Spionidae.

The analysis suggested that horned *Spiophanes* from the North East Atlantic and North Pacific represent two sister evolutionary lineages. The absence of *S*. *bombyx* from the Pacific, as previously suggested by Meißner & Blank [111], has been confirmed. Moreover, the present study suggested an even more complicated systematic composition of horned *Spiophanes* in the North East Atlantic. In contrast to Meißner & Blank [111], who suggested that *S*. *bombyx* was distributed in North European waters and in the Mediterranean Sea, we suggest that probably three sibling species: *S*. *bombyx*, *S*. cf. *bombyx* and *S*. cf. *convexus*, have been observed, and that *S*. *bombyx* probably occurs in the Mediterranean Sea only. Unfortunately, *S*. *convexus* was poorly described based on a few incomplete fragments, without consideration of the morphological variability of *S*. *bombyx* and not examined by molecular means. Molecular analysis of horned European *Spiophanes*, especially *S*. *bombyx* from the Gulf of Naples, is needed no clarify the species composition of this group in the region.

The analysis also revealed a complicated systematic composition of worms fitting the morphological characteristics of *S*. *kroyeri*. The discovery of two genetically different species in North European waters requires further clarification of their specific identity. Herein, based on the proximity of sampling sites of specimens examined in molecular analyses and the type localities of *S*. *kroyeri* and *S*. *cirrata*, we suggest that worms from the Barents and the Norwegian seas, and also from the northern part of the North Sea belong to *S*. *kroyeri*, whereas worms from the North Sea and the Bay of Biscay belong to *S*. *cirrata*. These suggestions should be verified by molecular examination of *Spiophanes kroyeri* from the Greenland Sea and in a broader-scale study of *Spiophanes* in North European waters.

The analysis also showed a rich species composition of horned *Spiophanes* in the North Pacific and all along the Pacific coast of North and South America. First of all, it confirmed that Asian and North American horned *Spiophanes*, even appearing morphologically identical, are not conspecific. Moreover, it showed that horned *Spiophanes* occurring along the Pacific coast of North America belong to two sibling species: *S*. *norrisi* and *S*. *hakaiensis* n. sp. The presence of sibling species among *Spiophanes* brings doubts about the conspecificity of remote populations in North and South America referred by Meißner & Blank [111] to *S*. *norrisi*. It is plausible that South American populations belongs to an undescribed sibling species.

### *COI* analysis, divergence time estimations and biogeographic inferences

The first Bayesian analysis of *COI* sequences, aiming to compare our data with those obtained in previous studies, resulted in a partially resolved consensus tree (Fig 1B), showing that short *COI* fragments (<300 bp) are not appropriate for these types of systematic inferences. The BEAST analysis of longer *COI* sequences (>500 bp) resulted in a fully resolved tree (Fig 3) with the same topology as in the five-genes Bayesian analysis (2B). A smaller set of taxa was used, however, in the BEAST analysis because it aimed to hypothesize on the divergence times of horned *Spiophanes* with metameric nuchal organs only.

Two hypotheses were addressed in advance to explain the high morphological similarity and disjunct distributions of horned *Spiophanes* on the Asian and American coasts of the North Pacific. Both hypotheses assumed that a common ancestor of those *Spiophanes* might have been distributed in the past all along the North Pacific without interruption. They differ however in the cause and the time of isolation of the two lineages, followed by subsequent speciation. The first hypothesis suggests that such isolation and speciation event might have resulted from the first opening of the Bering Strait at 5.5–5.4 mya [244, 245]. The second hypothesis links this isolation and speciation to the maximum glacial period in the Quaternary ice ages in the North Pacific 3.0–2.4 mya [246–250]. The estimate suggested by the BEAST analysis, about 1.3 mya (95% HPD: 2.0–0.7 mya), places the divergence of the North American and Asian lineages of horned *Spiophanes* after the maximum glacial period in the North Pacific and, thus, supports the second hypothesis. The last glacial maximum was suggested to have had a strong influence on the distribution of many other species (see Hewitt [251] for review).

Both the five-genes Bayesian analysis and the BEAST analysis suggested that the divergence of the North American and Asian lineages was preceded by the isolation and speciation of *S*. *norrisi* from a common North Pacific ancestor. The BEAST analysis estimates that this divergence might have happened about 1.7 mya (95% HPD: 2.3–1.0 mya). Remarkably, the boundary between the northern *S*. *hakaiensis* n. sp. and southern *S*. *norrisi* roughly coincides with the boundary between the Oregonian and Californian coastal biogeographical provinces at Point Conception [252]. Some Californian province species extend their ranges northward in warm-regime years [253–255]. This may also be the case with *S*. *norrisi* which occurs offshore from San Francisco, northern California.

The only other estimation of the divergence time of spionid polychaetes based on molecular data was provided by Schulze *et al*. [256] for North American populations of *Streblospio* spp. Using an invertebrate molecular clock calibration for *COI* sequence divergence of 1.8–2.8% per million years suggested by Palumbi [257], they estimated that the *Streblospio* lineage split in the Gulf of Mexico and adjacent regions 7–11 mya. The suggested estimates corresponded to the sea level minima in the examined region and this was considered as support for the obtained values of the estimates.

## Conclusions

The results of molecular analyses performed in the present study unambiguously showed the presence of a series of sibling species within the genus *Spiophanes*. It means that specific identifications of this complicated group of spionids in many cases should be verified by molecular data. Molecular identities of *Spiophanes*, especially from European waters, are needed for confident identifications of these polychaetes around the world.

## Supporting information

S1 Table List of samples reported in the present study. Sampling location data and museum registration numbers of newly collected material of *Spiophanes* spp.; museum samples examined during this study; material from the Norwegian, North, Mediterranean and Aegean seas and North Pacific reported by Meißner & Blank [111] and from the North Sea identified by Dieter Fiege, Markus Böggemann and Karin Meißner which is deposited in the SMF and other polychaete collections and was only partially re-examined by the first author (VIR); records of *S*. *bombyx* (= *S*. *uschakowi*) from Japan provided by Imajima [88, 116–118], for which no museum numbers were reported; records of *S*. *bombyx* (= *S*. cf. *norrisi*) from Chile, Argentina and the Falkland Islands provided by Carrasco [119] and Blake [48]; records of *S*. *bombyx* (here referred either to *S*. *bombyx* or *S*. cf. *convexus*) from Spain provided by Meißner [8].
(XLSX)

S2 Table List of the museums and collections (and their acronyms) holding the examined or reported samples of Spiophanes species. (XLSX)

S3 Table Genetic distances between *Spiophanes* species—*COI*. Uncorrected pairwise average distances (*p*, in %) between specific clades for *COI* (534 bp) sequences used in the five-genes Bayesian analysis of *Spiophanes* spp. rooted with sequences of *Trochochaeta multisetosa*. The numbers of corresponding samples from Table 1 are given in parentheses after names and sampling locations of the species. Average distances between sympatric conspecific individuals in italics, maximal values of average distances between different *Spiophanes* species in bold, and minimal values of average distances between different *Spiophanes* species underlined.
(XLSX)

S4 Table Genetic distances between *Spiophanes* species– *16S*. Uncorrected pairwise average distances (*p*, in %) between specific clades for *16S* (244 bp) sequences used in the five-genes Bayesian analysis of *Spiophanes* spp. rooted with sequences of *Trochochaeta multisetosa*. The numbers of corresponding samples from Table 1 are given in parentheses after names and sampling locations of the species. Average distances between sympatric conspecific individuals in italics, maximal values of average distances between different *Spiophanes* species in bold, and minimal values of average distances between different *Spiophanes* species underlined.
(XLSX)

S5 Table Genetic distances between *Spiophanes* species– *18S*. Uncorrected pairwise average distances (*p*, in %) between specific clades for *18S* (1656 bp) sequences used in the five-genes Bayesian analysis of *Spiophanes* spp. rooted with sequences of *Trochochaeta multisetosa*. The numbers of corresponding samples from Table 1 are given in parentheses after names and sampling locations of the species. Average distances between sympatric conspecific individuals in italics, maximal values of average distances between different *Spiophanes* species in bold, and minimal values of average distances between different *Spiophanes* species underlined.
(XLSX)

S6 Table Genetic distances between *Spiophanes* species– *28S*. Uncorrected pairwise average distances (*p*, in %) between specific clades for *28S* (287 bp) sequences used in the five-genes Bayesian analysis of *Spiophanes* spp. rooted with sequences of *Trochochaeta multisetosa*. The numbers of corresponding samples from Table 1 are given in parentheses after names and sampling locations of the species. Average distances between sympatric conspecific individuals in italics, maximal values of average distances between different *Spiophanes* species in bold, and minimal values of average distances between different *Spiophanes* species underlined.
(XLSX)

S7 Table Genetic distances between *Spiophanes* species–*Histone 3*. Uncorrected pairwise average distances (*p*, in %) between specific clades for *Histone 3* (297 bp) sequences used in the five-genes Bayesian analysis of *Spiophanes* spp. rooted with sequences of *Trochochaeta multisetosa*. The numbers of corresponding samples from Table 1 are given in parentheses after names and sampling locations of the species. Average distances between sympatric conspecific individuals in italics, maximal values of average distances between different *Spiophanes* species in bold, and minimal values of average distances between different *Spiophanes* species underlined.
(XLSX)
